# Amorphous AlPO_4_ Layer Coating Vacuum Thermal Reduced SiO*_x_* with Fine Silicon Grains to Enhance the Anode Stability

**DOI:** 10.1002/advs.202405116

**Published:** 2024-07-30

**Authors:** Jingyi Luan, Hongyan Yuan, Jie Liu, Naiqin Zhao, Wenbin Hu, Cheng Zhong

**Affiliations:** ^1^ Key Laboratory of Advanced Ceramics and Machining Technology (Ministry of Education) Tianjin Key Laboratory of Composite and Functional Materials School of Materials Science and Engineering Tianjin University Tianjin 300072 China; ^2^ Joint School of National University of Singapore and Tianjin University International Campus of Tianjin University Binhai New City Fuzhou 350207 China

**Keywords:** amorphous AlPO_4_ layer, fine silicon grains, lithium ion batteries, vacuum thermal reduction

## Abstract

Micrometer‐sized silicon monoxide (SiO) is regarded as a high‐capacity anode material with great potential for lithium ion batteries (LIBs). However, the problems of low initial Coulombic efficiency (ICE), poor electrical conductivity, and large volume change of SiO inevitably impede further application. Herein, the vacuum thermal reduced SiO*_x_* with amorphous AlPO_4_ and carbon double‐coating layers is used as the ideal anode material in LIBs. The vacuum thermal reduction at low temperature forms fine silicon grains in the internal particles and maintains the external integrity of SiO*_x_* particles, contributing to mitigation of the stress intensification and the subsequent design of multifunctional coating. Meanwhile, the innovative introduction of the multifunctional amorphous AlPO_4_ layer not only improves the ion/electron conduction properties to ensure the fast reversible reaction but also provides a robust protective layer with stable physicochemical characteristics and inhibits the volume expansion effect. The sample of SiO*_x_* anode shows an ICE up to 87.6% and a stable cycling of 200 cycles at 1 A g^−1^ with an initial specific capacity of 1775.8 mAh g^−1^. In addition, the assembled pouch battery of 1.8 Ah can also ensure a cycling life of over 150 cycles, demonstrating a promising prospect of this optimized micrometer‐sized SiO*_x_* anode material for industrial applications.

## Graphical abstract

The vacuum thermal reduction at low temperature ensures structural integrity of SiO*_x_* and uniform distribution of fine silicon grains inside, contributing to mitigation of expansion stress. Moreover, the multifunctional amorphous AlPO_4_ layer with high Young's modulus not only controls the plastic deformation of SiO*_x_* during the cycling process but also improves ion/electron conduction properties to ensure the fast reversible reactions.

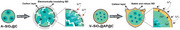

## 1 Introduction

The growing demands for electric vehicles and portable electronics for reducing the charging frequency require batteries to work for a longer time after being fully charged, which has stimulated the in‐depth study of lithium ion batteries (LIBs) with high energy density.^[^ ^1^ ^]^ The commercial graphite anode is a kind of well‐established material with the limited specific capacity of 372 mAh g^−1^, which makes it hard to achieve the requirements of high energy density LIBs.^[^ ^2^ ^]^ Therefore, it is important to explore novel anode materials with higher specific capacity. Micrometer‐sized silicon monoxide (SiO) is considered as a promising alternative because of the advantages of low cost, high tap density, and high theoretical capacity (≈2400 mAh g^−1^).^[^ ^3^ ^]^ Nevertheless, some issues still hinder the practical application of SiO in LIBs, such as low initial Coulombic efficiency (ICE) (≈50%), huge volume expansion of SiO (≈200%), and poor intrinsic electrical conductivity.^[^ ^4^ ^]^

Low ICE is the primary limitation of SiO anode, which causes excessive consumption of limited lithium ions for the first cycle, requiring more active cathode material and being non‐conducive to increasing the energy density of the full cell.^[^ ^5^ ^]^ The fundamental reason for the low ICE is that SiO material reacts with lithium ions to form the irreversible phase of Li_2_O and lithium silicates during the first lithiation process, which is electrochemically inert and does not participate in electrode reactions during subsequent cycles.^[^ ^6^ ^]^ Therefore, reducing the stoichiometric ratio of oxygen in SiO*_x_* (0 < *x* < 1) is an effective method to improve the ICE of SiO*_x_* anode.^[^ ^7^ ^]^ The strategy of magnesiothermic reduction is usually used to consume partial oxygen and increase the active silicon phase ratio in SiO*_x_*, which has the merits of low cost and simple process.^[^ ^8^ ^]^ For example, Lee et al.^[^ ^9^ ^]^ synthesized SiO_x_/Mg_2_SiO_4_/SiO*_x_* composite via the magnesiothermic reduction method, and they found that the ICE of the composite was gradually improved with the increase of Mg molar ratio. When the molar ratios of Mg were 0.5, 0.7, and 0.9, the corresponding ICE increased to 75.6%, 78.5%, and 86.8 respectively, compared with 56.6% of pristine SiO. However, the direct mixing of SiO and Mg powder always resulted in the uneven reduction of SiO. The local violent reduction reaction would result in an excessively high local temperature and damage the morphology of SiO particles, which would accelerate the growth of silicon grains inside SiO*_x_* and cause the increasing formation of solid electrolyte interface (SEI). In order to ensure a uniform reaction process, Lim et al.^[^ ^7^ ^]^ prepared Mg‐SiO*_x_* particles via a physical vapor deposition method at 1 Torr and 1400 °C. Under the condition of optimal Mg concentration, the homogeneous distribution of silicon clusters was observed, and an ICE of 79.9% and specific capacity of 1286.4 mAh g^−1^ had been achieved. Unfortunately, the high experiment temperatures and equipment costs make this approach relatively difficult to be scalable.

The large volume expansion and poor intrinsic electrical conductivity of SiO anode are also challenges that need to be overcome. Researchers have attempted to design multifunctional coatings on the surface of SiO*_x_* to facilitate ion/electron transport and suppress volume expansion. For example, the Al_2_O_3_ layer was used as an ionically conductive coating to reduce the formation of SEI and stabilize the structure of the Si‐based anode.^[^ ^10^ ^]^ In addition, carbon coating is also a useful approach to optimize the transport rate of electrons and accommodate the volume expansion of SiO*_x_* anode due to the high conductivity and superior resilience of carbon materials.^[^ ^11^ ^]^ For example, Liu et al.^[^ ^12^ ^]^ modified disproportionated SiO microparticles coated with vertical graphene nanosheets (d‐SiO@vG). The d‐SiO@vG anode demonstrated a specific capacity of 1600 mAh g^−1^ and remained stable over 100 cycles with a retention of 93%. To sum up, the combination of various functional coatings is expected to be a promising strategy for enhancing the stability of SiO_x_ anode.

In this work, we utilized a vacuum magnesiothermic reduction method with low energy consumption to enhance the ICE of SiO*_x_* and prepare the amorphous AlPO_4_ and carbon double‐coating layers to reduce the volume expansion and improve the electrical conductivity of the SiO*_x_* anode. Compared to the vacuum calcination methods with the temperature more than 1300 °C reported in the literatures, the vacuum calcination process at relatively low temperature of 750 °C could reduce energy consumption and guarantee a mild reaction between SiO and Mg, thereby maintaining the integrity of SiO*_x_* particles.^[^ ^7^, ^13^ ^]^ The intact particles did not only reduce the consumption of active substances and electrolytes due to the formation of SEI and contribute to the mitigation of the stress intensification owing to large volume expansion but also preserved enough space for the design of multifunctional coatings in the structure.^[^ ^14^ ^]^ Meanwhile, reducing SiO by Mg vapor could promote the homogeneity of the reaction and prepare SiO*_x_* with fine silicon grains, which was beneficial to relieve the volume expansion and contraction upon repeated cycling. However, it cannot be ignored that the increase of Si ratio also made the volume expansion effect more obvious, compared with the original SiO. The introduction of multifunctional coatings has effectively solved the above problems. The formed AlPO_4_ protection layer has strong covalent bonds with high Young's modulus, which can effectively control the plastic deformation of SiO*_x_* and alleviate the parasitic reactions during the cycling process. In addition, the carbon coating method of chemical vapor deposition using CH_4_ as the carbon source can ensure the uniform thickness of the coating layer outside each particle, which is conducive to improving the electrode reaction kinetics and reducing the volume expansion. In addition, the amorphous AlPO_4_ and carbon double‐coating layers can build a robust and fast‐ion conducting interphase to improve the electron transfer rate and the storage capability of lithium ions. Therefore, the as‐prepared V–SiO*_x_* @AP@C delivers an ultrahigh ICE of 87.6% and an initial specific capacity of 1775.8 mAh g^−1^, and the corresponding pouch battery of 1.8 Ah can maintain stable cycling over 150 cycles at 0.5C. This research provides a promising strategy to acquire high‐performance micrometer‐sized SiO*_x_* anode with much smaller volume expansion and superior cycling reversibility in LIBs.

## 2 Results and Discussion

**Figure 1:**
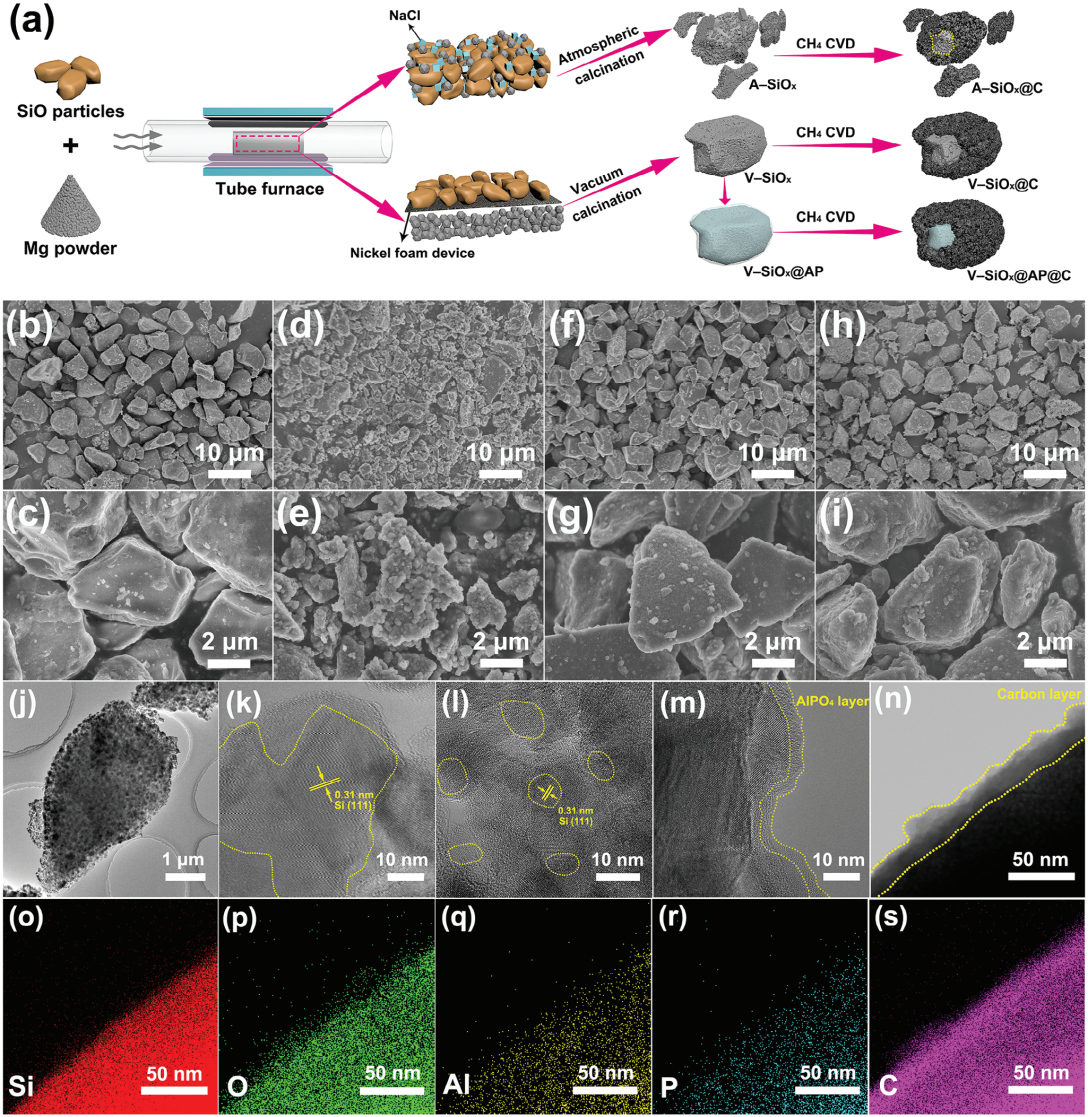
a) Schematic illustration of the preparation process of A–SiO*_x_* @C, V–SiO*_x_* @C, and V–SiO*_x_* @AP@C. SEM images of b,c) SiO, d,e) A–SiO*_x_* @C, f,g) V–SiO*_x_* @C, and h,i) V–SiO*_x_* @AP@C at different magnifications. j) TEM image of V–SiO*_x_*. HRTEM images of k) A–SiO*_x_*, l) V–SiO*_x_*, and m) V–SiO*_x_* @AP. n) TEM image of V–SiO*_x_* @AP@C and o–s) the corresponding elemental mapping images of Si, O, Al, P, and C.

With respect to comparing different magnesiothermic reduction methods, **Figure** **1**a illustrates the preparation processes of thermal reduced SiO*_x_* under atmosphere pressure (denoted as A–SiO*_x_*) and vacuum thermal reduced SiO*_x_* (denoted as V–SiO*_x_*). V–SiO*_x_* is then further optimized by coating the AlPO_4_ layer (denoted as V–SiO*_x_* @AP). To improve the electrical conductivity and mitigate the effects of volume expansion, all the samples are coated with carbon layers by the process of subsequent chemical vapor deposition (CVD), which are denoted as A–SiO_*x*_@C,V–SiO_*x*_@C and V–SiO_*x*_@AP@C, respectively. The morphologies and size distributions of samples are investigated with scanning electron microscopy (SEM), as exhibited in Figure 1b–i. As for the raw material of micrometer‐sized SiO, the corresponding particle size is uniform and ≈5 µm (Figure 1b) and the surface of SiO particles is smooth (Figure 1c; Figure S1a, Supporting Information). After the magnesiothermic reduction process under atmosphere pressure, the particles of SiO*_x_* are seriously destroyed and form fragments of different sizes with rough surfaces (Figure 1d,e), which is caused by the local violent reduction reaction due to the direct mixing of SiO and Mg powder in A–SiO*_x_* @C. In contrast, V–SiO*_x_* @C prepared by vacuum thermal reduction effectively maintains the integrity of particles (Figure 1f,g) owing to the homogenous contact and reaction of Mg vapor and SiO. The integrity of V–SiO*_x_* @C particles is beneficial to mitigation of the stress intensification resulting from the large volume expansion and the subsequent design of multifunctional coating. Further, from the magnified SEM images, the large spherical protrusions formed on the surface of A–SiO*_x_* @C (Figure S1b, Supporting Information) are observed owing to excessive local reaction temperature. In comparison, tiny particles distribute evenly on the surface of V–SiO*_x_* @C (Figure S1c, Supporting Information), which is conductive to the uniform distribution of the electric field and the growth of stable SEI. As can be seen in Figure 1h,i, V–SiO*_x_* @AP@C still maintains the superiority of V–SiO*_x_* @C in that the particles are homogenous and intact after the introduction of the AlPO_4_ layer. Meanwhile, the details of V–SiO*_x_* @AP@C particles are exhibited in Figure S1d (Supporting Information), in which the tiny particles on the surface become less obvious due to the coating of the AlPO_4_ layer. The corresponding elemental mapping images of Si, O, Al, and P in V–SiO*_x_* @AP are captured in Figure S2 (Supporting Information), which proves the homogeneous distribution of the AlPO_4_ layer.

To further investigate the internal structure and composition of different samples, transmission electron microscope (TEM) analysis is also performed. Compared to the pristine SiO with intact and dense structure (Figure S3a, Supporting Information), the particles of A–SiO*_x_* are broken seriously with blurred boundaries, which are composed of fragments with different sizes (Figure S3b, Supporting Information). In contrast, V–SiO*_x_* maintains the great morphological integrity of particles (Figure 1j), which further demonstrates the superiority of the mild and uniform vacuum thermal reduction method. Meanwhile, the internal nanoparticles and pores are evenly distributed in V–SiO*_x_*, contributing to alleviating the effects of severe volume expansion and particle cracking during the cycling process. Besides, high‐resolution transmission electron microscopy (HRTEM) images further reveal the growth of silicon grains in samples. This is because the local violent reaction of Mg powder and SiO results in an excessively high local temperature, which accelerates the growth of silicon grains inside SiO*_x_*. In Figure 1k and S4a (Supporting Information), the silicon grain size of A–SiO*_x_* is large with clear lattices, which may lead to large volume change during the lithiation/delithiation process and result in the structure collapse. In contrast, in the preparation process of V–SiO*_x_*, the homogenous contact of Mg vapor and SiO may promote the homogeneity of the reaction and thus the distribution of reaction temperature could be uniform. Excitingly, Figure 1l and S4b (Supporting Information) exhibit that the fine silicon grains are evenly embedded in the V–SiO*_x_* and surrounded by the amorphous SiO*_x_* phases, which can be regarded as a buffer to accommodate the volume change of particles during the cycling process. With the subsequent introduction of amorphous AlPO_4_ and carbon coatings, it can be observed that surfaces of V–SiO*_x_* @AP and V–SiO*_x_* @AP@C are coated with the thin amorphous AlPO_4_ layer (Figure 1m) and obvious carbon coating (Figure 1n; Figure S5, Supporting Information), respectively. In addition, as exhibited in Figure 1o–s, the corresponding elements of Si, O, Al, P, and C are uniformly distributed in V–SiO*_x_* @AP@C, further proving the successful construction of multifunctional coating.

**Figure 2:**
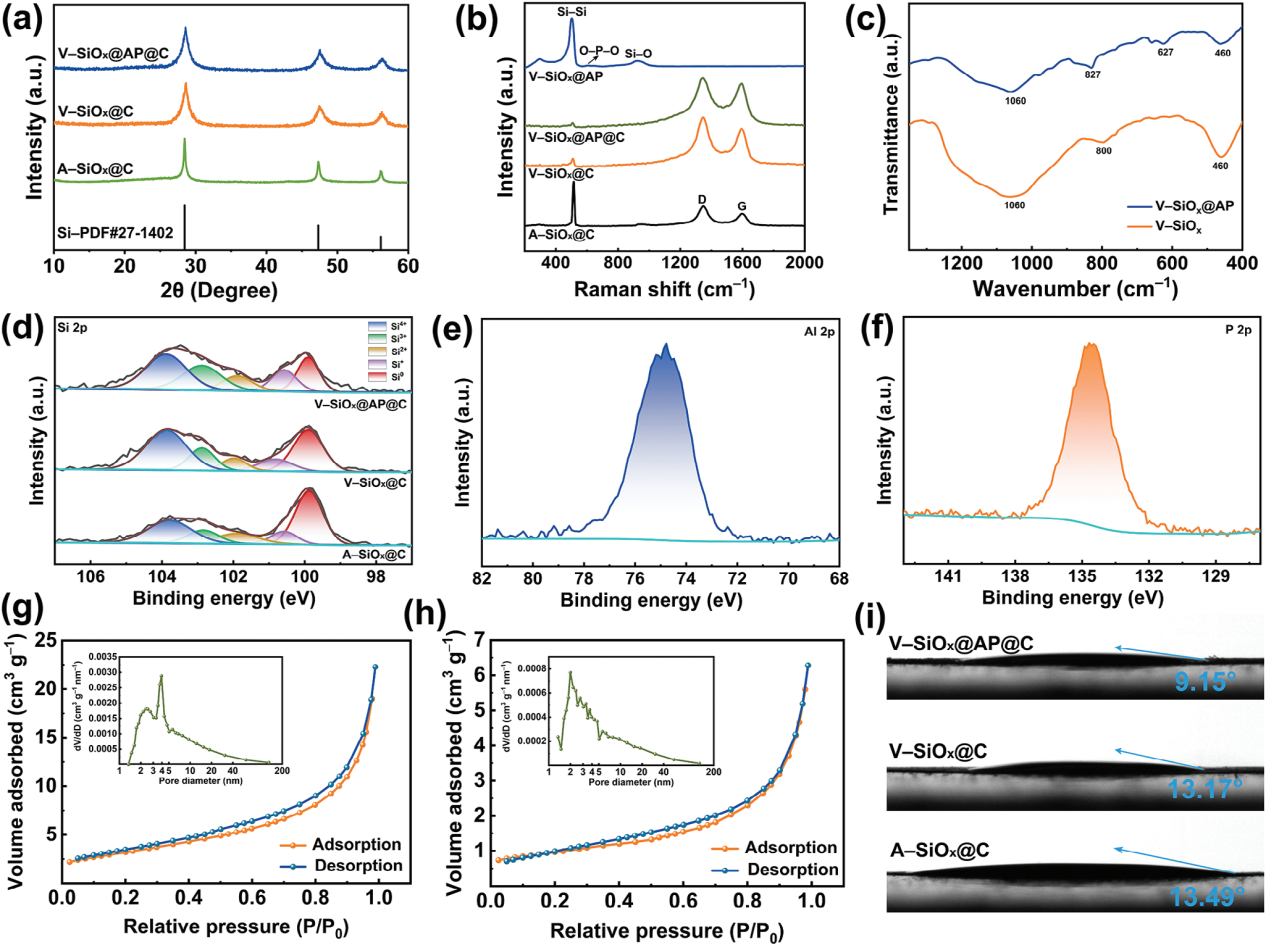
a) XRD patterns of A–SiO*_x_* @C, V–SiO*_x_* @C, and V–SiO*_x_* @AP@C. b) Raman spectra of A–SiO*_x_* @C, V–SiO*_x_* @C, V–SiO*_x_* @AP@C, and V–SiO_x_@AP. c) FTIR curves of V–SiO*_x_* and V–SiO*_x_* @AP. d) XPS spectra of Si 2p of A–SiO*_x_* @C, V–SiO*_x_* @C, and V–SiO*_x_* @AP@C. XPS spectra of e) Al 2p and f) P 2p of V–SiO*_x_* @AP. N_2_ adsorption–desorption isotherms and the corresponding pore size distribution (inset) of g) A–SiO*_x_* @C and h) V–SiO*_x_* @AP@C. i) Contact angles of A–SiO*_x_* @C, V–SiO*_x_* @C, and V–SiO*_x_* @AP@C.

X‐ray diffraction (XRD) is carried out to evaluate the phase evolution of samples. As demonstrated in Figure S6 (Supporting Information), there is a wide peak of ≈23° of SiO, depicting the characteristic of the amorphous nature. In **Figure** **2**a, the weak peaks in the range of 20–25° are indexed with the amorphous phase of silicon oxide, and the peaks located at 28.4°, 47.3°, and 56.1° match well with the standard diffraction peaks of Si (JCPDS No. 27–1402). Moreover, the full width‐half‐maximum values of Si peaks in V–SiO*_x_* @C and V–SiO*_x_* @AP@C are much wider than those of A–SiO*_x_* @C, demonstrating the smaller crystalline Si size.^[^ ^7^ ^]^ According to the Scherrer equation, the average sizes of crystalline Si grains inside A–SiO*_x_* @C, V–SiO*_x_* @C, and V–SiO*_x_* @AP@C are 18.08, 7.06, and 7.07 nm, respectively, which correspond to the results illustrated in TEM images.^[^ ^15^ ^]^ The samples are further characterized by Raman spectroscopy and corresponding results are demonstrated in Figure 2b. The sharp peaks located at 510 cm^−1^ are attributed to Si─Si bonds, and the wide peak at 936 cm^−1^ is indexed with Si─O bonds in amorphous SiO*_x_*.^[^ ^16^ ^]^ Meanwhile, the broad peaks distributed at 1350 and 1600 cm^−1^ match with the disordered D‐band and the ordered graphic G‐band of carbon, respectively.^[^ ^17^ ^]^ The intensity of D bands of A–SiO*_x_* @C, V–SiO*_x_* @C, and V–SiO*_x_* @AP@C is higher than that of G bands, demonstrating a high disordered degree of carbon layers, which contributes to the transport of lithium ions due to the isotropic feature.^[^ ^18^ ^]^ The sample of V–SiO*_x_* @AP is used to characterize the thin coating layer of amorphous AlPO_4_ because the thick carbon coating can affect the test accuracy. The peak at 617 cm^−1^ belongs to the O─P─O bending mode, demonstrating the existence of AlPO_4_.^[^ ^19^ ^]^ The comparison of Fourier transform infrared (FTIR) curves of V–SiO*_x_* and V–SiO*_x_* @AP further testifies to the successful introduction of amorphous AlPO_4_ into V–SiO*_x_* @AP. The peaks at 1060, 800/827, and 460 cm^−1^ of V–SiO*_x_* and V–SiO*_x_* @AP are assigned to the Si─O─Si bands.^[^ ^20^ ^]^ In addition, the peak at 627 cm^−1^ of V–SiO*_x_* @AP corresponds to the asymmetric and symmetric stretching vibrations of the Al─O─P units.^[^ ^21^ ^]^

X‐ray photoemission spectroscopy (XPS) of Si 2p is conducted to analyze the valence states of Si of samples (Figure 2d). The peaks are assigned to Si^0^ (99.8 eV), Si^+^ (100.6 eV), Si^2+^ (101.9 eV), Si^3+^ (102.8 eV), and Si^4+^ (103.9 eV), respectively.^[^ ^4a^ ^]^ The relative intensity of peaks of Si^+^, Si^2+^, Si^3+^, and Si^4+^ in V–SiO*_x_* @C and V–SiO*_x_* @AP@C is higher than those of A–SiO*_x_* @C, indicating a wider distribution of amorphous SiO*_x_*, which can help to relieve the stress of volume expansion. In addition, as shown in Figure S7 (Supporting Information), the characteristic peaks of Si 2p, O 1s, Al 2p, and P 2p appear in the XPS survey spectrum. In addition, Al 2p at 74.9 eV (Figure 2e) and P 2p at 134.6 eV (Figure 2f) further verify that the AlPO_4_ layer is successfully coated on the surface of V–SiO*_x_*.^[^ ^22^ ^]^ Subsequently, Brunauer–Emmett–Teller (BET) analysis is carried out to study the porous structure of the samples. All the samples exhibit the characteristic of the type IV adsorption isotherms with an H3 hysteresis loop (Figure 2g,h; Figure S8, Supporting Information), indicating the porous structures. The surface areas of pristine SiO, A–SiO*_x_* @C, V–SiO*_x_* @C, and V–SiO*_x_* @AP@C are 1.45, 13.54, 4.69, and 4.02 m^2^ g^−1^, respectively. The high surface area of A–SiO*_x_* @C conforms to the characteristic of broken fragments of different sizes as shown in the above SEM images. In comparison, V–SiO*_x_* @AP@C shows a moderate surface area because the vacuum thermal reduction maintains the integrity of particles well, which is beneficial for improving the cycling stability. Further, the corresponding total pore volumes of pristine SiO, A–SiO*_x_* @C, V–SiO*_x_* @C, and V–SiO*_x_* @AP@C are 0.003, 0.035, 0.012, and 0.010 cm^3^ g^−1^, respectively. The expanded pore volumes of modified samples can provide sufficient transmission channels for lithium ions and better adapt to volume changes. In addition, sufficient pore structures enhance the wettability of modified samples, which have small contact angles (Figure 2i), facilitating the adsorption of the electrolyte. Particularly, the contact angle of V–SiO*_x_* @AP@C is the smallest, which may be due to the coexistence of cations and framework anion sites of AlPO_4_ forming an electrostatic field that further promotes the affinity of V–SiO*_x_* @AP@C to the electrolyte.^[^ ^23^ ^]^

**Figure 3:**
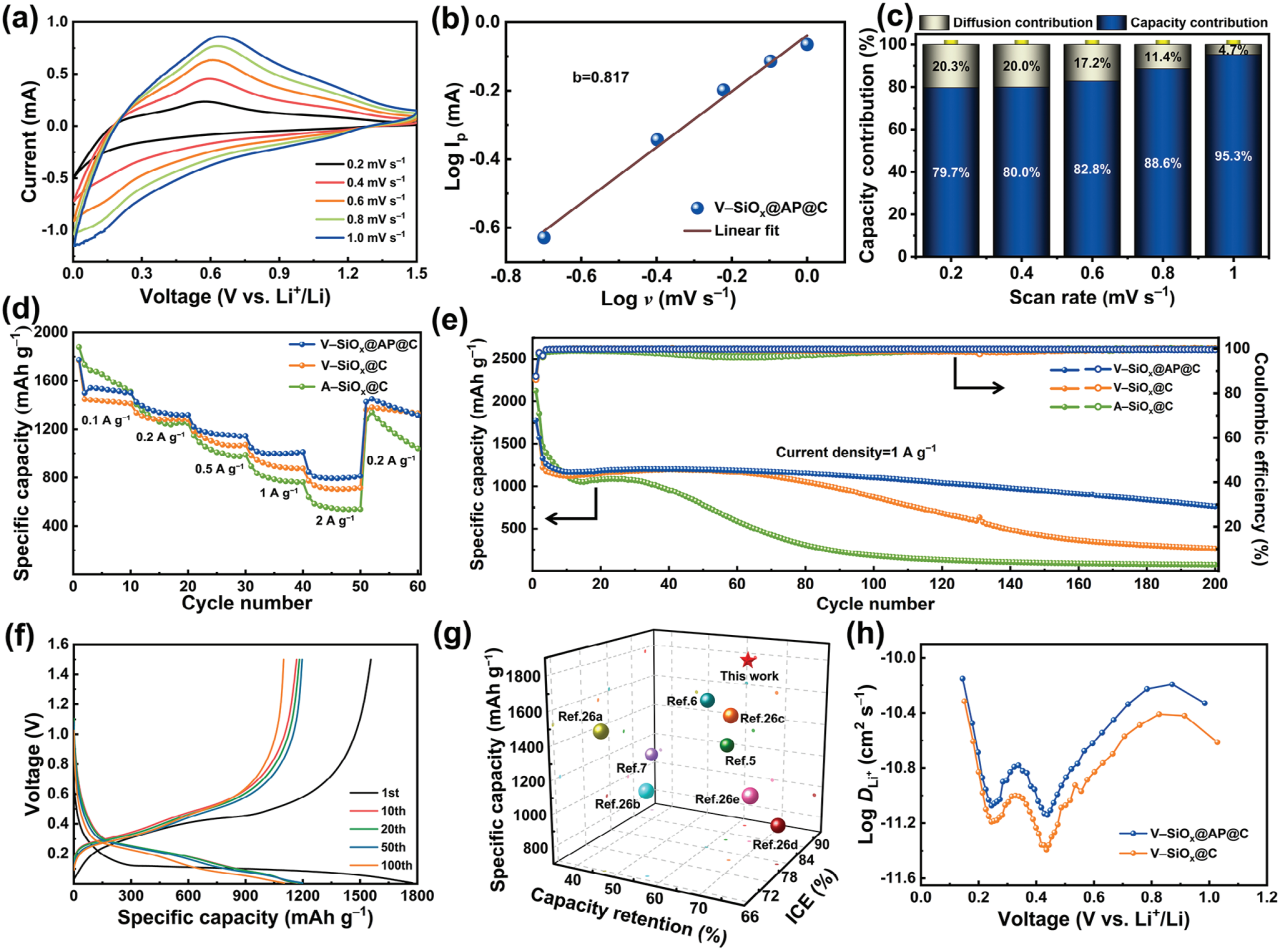
a) CV curves of V–SiO*_x_*_@_AP@C at different scanning rates. b) Logarithm plots of anodic peak currents of V–SiO*_x_* @AP@C versus scan rates. c) Percentage capacitive contributions of V–SiO*_x_* @AP@C at different scan rates. d) Rate performance of A–SiO*_x_* @C, V–SiO*_x_* @C, and V–SiO*_x_* @AP@C from 0.1 to 2 A g^−1^. e) Long‐term cycling performance of A–SiO*_x_* @C, V–SiO*_x_* @C, and V–SiO*_x_* @AP@C (the current density of the first two activated cycles is 137.5 mA g^−1^ and the current density of the subsequent cycles is 1 A g^−1^). f) Charge/discharge profiles of V–SiO*_x_* @AP@C with different cycles (the current density of the first two activated cycles is 137.5 mA g^−1^ and the current density of the subsequent cycles is 1 A g^−1^). g) Comparison of the performance of V–SiO*_x_* @AP@C with previously reported SiO*_x_*‐based anode materials in LIBs. h) $D_{Li^{+}}$ against voltage for the delithiation process of V–SiO*_x_* @C and V–SiO*_x_* @AP@C via GITT measurement.

The electrochemical performances of the prepared samples are investigated by assembling coin cells. As shown in cyclic voltammetry (CV) curves (Figure S9, Supporting Information), during the first lithiation process, the peaks at ≈0.7–0.8 V and 1.2–1.3 V are indexed with the formation of the SEI film, which disappears in the following cycles. Moreover, a conspicuous reduction peak at 0.1–0.2 V is indexed with the phase transition from crystalline Si to amorphous Li*_x_* Si. Upon delithiation, the peaks at ≈0.37 and 0.53 V correspond to the dealloying process of Li*_x_* Si into amorphous Si. In addition, the current intensities of corresponding redox peaks gradually increase with the subsequent cycles due to the enhancement of active reactions between the active materials and lithium ions. The current density in CV curves of V–SiO*_x_* @AP@C is higher than those of A–SiO*_x_* @C and V–SiO*_x_* @C, demonstrating the greater electrochemical activity of V–SiO*_x_* @AP@C. To investigate the kinetic behavior of the electrodes, CV tests at different scan rates are carried out (**Figure** **3**a; Figure S10, Supporting Information). The capacitive effect can be qualitatively evaluated by Equations (1) and (2) between peak current (*i*) and scan rate (*ʋ*):

(1) $$i=av^{b}$$

(2) logi=blogv+loga

where *a* and *b* represent two adjustable parameters.^[^ ^24^ ^]^ *b* = 0.5 demonstrates that charge storage kinetics is controlled by a diffusion process, while *b* = 1 suggests that charge storage kinetics is controlled via a capacitive process. In Figure 3b and S11 (Supporting Information), the acquired *b* values for the anodic peaks of A–SiO*_x_* @C, V–SiO*_x_* @C, and V–SiO*_x_* @AP@C are 0.797, 0.812, and 0.817, respectively, demonstrating that the electrochemical reactions exhibit typical pseudocapacitive characteristics. The percentage capacitive contributions at different scan rates are calculated based on the following Equation (3):

(3) $$i=k_{1}v+k_{2}v^{1/2}$$

where *k*_1_ *v* is the ratio of capacitive contribution and *k*_2_ *v* ^1/2^ is the ratio of diffusion contribution.^[^ ^24^, ^25^ ^]^ Figure S12 (Supporting Information) illustrates the capacitive contributions of A–SiO*_x_* @C, V–SiO*_x_* @C, and V–SiO*_x_* @AP@C at 1 mV s^−1^. As observed in Figure 3c and S13 (Supporting Information), the capacitive contribution gradually increases with the increases of scan rate. The capacitive contribution of V–SiO*_x_* @AP@C rise from 79.7% at 0.2 mV s^−1^ to 95.3% at 1 mV s^−1^, higher than those of A–SiO*_x_* @C and V–SiO*_x_* @C. According to the *b* value and the ratios of capacitive contribution in the above kinetic analysis, it is illustrated that the majority of charge storage in V–SiO*_x_* @AP@C derives from the pseudocapacitive processes, which is conducive to the fast charge storage and long‐term cycling ability.^[^ ^26^ ^]^

As rate performance determines the output power, it is necessary to test the cycling performance at different current densities (Figure 3d). Obviously, V–SiO*_x_* @AP@C demonstrates high discharge capacity and more stable capacity retention at various current densities compared with those of A–SiO*_x_* @C and V–SiO*_x_* @C. This is because introduction of the amorphous AlPO_4_ can strengthen the interfacial kinetics of the charge carrier (lithium ions/electrons), which effectively improves the discharge capacity and the electrochemical reaction reversibility. Moreover, the long‐term cycling performance and corresponding Coulombic efficiency of the samples are tested at 1 A g^−1^ and shown in Figure 3e. Although A–SiO*_x_* @C has a higher discharge capacity compared with those of V–SiO*_x_* @C and V–SiO*_x_* @AP@C in the initial cycle, A–SiO*_x_* @C suffers a drastic capacity fading after 40 cycles and decreases to a low discharge capacity of 183.6 mAh g^−1^ at the 100th cycle. In contrast, V–SiO*_x_* @C exhibits more stable cycling performance with a discharge capacity of 265.3 mAh g^−1^ after 200 cycles due to the advantage of vacuum thermal reduction at low temperature. By the further optimization of the amorphous AlPO_4_ layer, V–SiO*_x_* @AP@C has superior stable cycling with a high capacity retention of 761.5 mAh g^−1^ after 200 cycles. Figure S14 (Supporting Information) exhibits the charge/discharge profiles of A–SiO*_x_* @C and V–SiO*_x_* @C at different cycles. The discharge capacity of A–SiO*_x_* @C can remain stable without obvious fading before 20 cycles but decreases after the subsequent cycles. This is because the repeated insertion/extraction of lithium ions will aggravate the volume expansion of anodes with the extension of cycles, causing active materials pulverization and exfoliation from the current collectors. In contrast, V–SiO*_x_* @C maintains stable discharge capacity until 50 cycles because the internal silicon fines generated by vacuum thermal reduction at low temperature can reduce the volume expansion. Satisfyingly, the capacity decay of V–SiO*_x_* @AP@C is not obvious during the first 100 cycles (Figure 3f), which is due to the construction of robust and fast‐ion conducting interphase of amorphous AlPO_4_. In practical application, high ICE is critical for anode materials because anodes with low ICE require more mass of cathode materials to match, which is not conducive to improving the energy density of the full battery. V–SiO*_x_* @AP@C demonstrates a relatively high ICE of 87.6% with a high initial discharge capacity of 1775.8 mAh g^−1^. Figure 3g provides a performance comparison of V–SiO*_x_* @AP@C with the SiO*_x_*‐based anode materials in LIBs reported in recent years, which proves the superiority of V–SiO*_x_* @AP@C in ICE, and initial discharge capacity and capacity retention, showing excellent practical application potential.^[^ ^5^, ^6^, ^7^, ^27^ ^]^ The corresponding detailed information is shown in Table S1 (Supporting Information).

The dynamic behavior of V–SiO*_x_* @C and V–SiO*_x_* @AP@C are investigated by galvanostatic intermittent titration technique (GITT) test shown in Figure S15 (Supporting Information). According to Fick's second law, the diffusion coefficient of Li^+^ ($D_{Li^{+}}$) is calculated by the following Equation (4):

(4) $$D_{Li^{+}}=\frac{4}{\pi \tau }\;{\left(\frac{m_{B}V_{M}}{M_{B}S}\right)}^{2}{\left(\frac{\Delta E_{s}}{\Delta E_{\tau }}\right)}^{2}$$

where *m*_B_, *V*_M_, *M*_B_, and *S* indicate the active mass, molar volume, molar mass, and effective surface area of the electrode, respectively.^[^ ^28^ ^]^ The parameters of τ (the galvanostatic current time), ∆*E*_s_ (the steady‐state voltage change), and ∆*E*_τ_ (the total transient voltage change) can be obtained from the relation curves between potential and charge–discharge time in the GITT test. As exhibited in Figure 3h and S16 (Supporting Information), the $D_{Li^{+}}$ of V–SiO*_x_* @AP@C is always higher than that of V–SiO*_x_* @C, which illustrates that the introduction of amorphous AlPO_4_ can effectively promote the diffusion rate of lithium ion and optimize the kinetic behavior. Further, the electrochemical impedance spectra (EIS) of the samples after 10 and 200 cycles are obtained (Figure S17, Supporting Information), which are used to investigate the lithium ion migration resistance through the interface by the comparison of the diameters of the semicircles in the high frequency regions. According to the results,V–SiO*_x_* @AP@C exhibits the smallest semicircle among the samples after 10 cycles, which may be due to the high conductivity of AlPO_4_ coating. After 200 cycles, the total interfacial resistance of A–SiO*_x_* @C increases significantly, revealing that the SEI is unstable due to the structure degradation. By contrast, the interfacial resistance of V–SiO*_x_* @AP@C is almost unchanged, demonstrating the superior stability of interface and structure.

**Figure 4:**
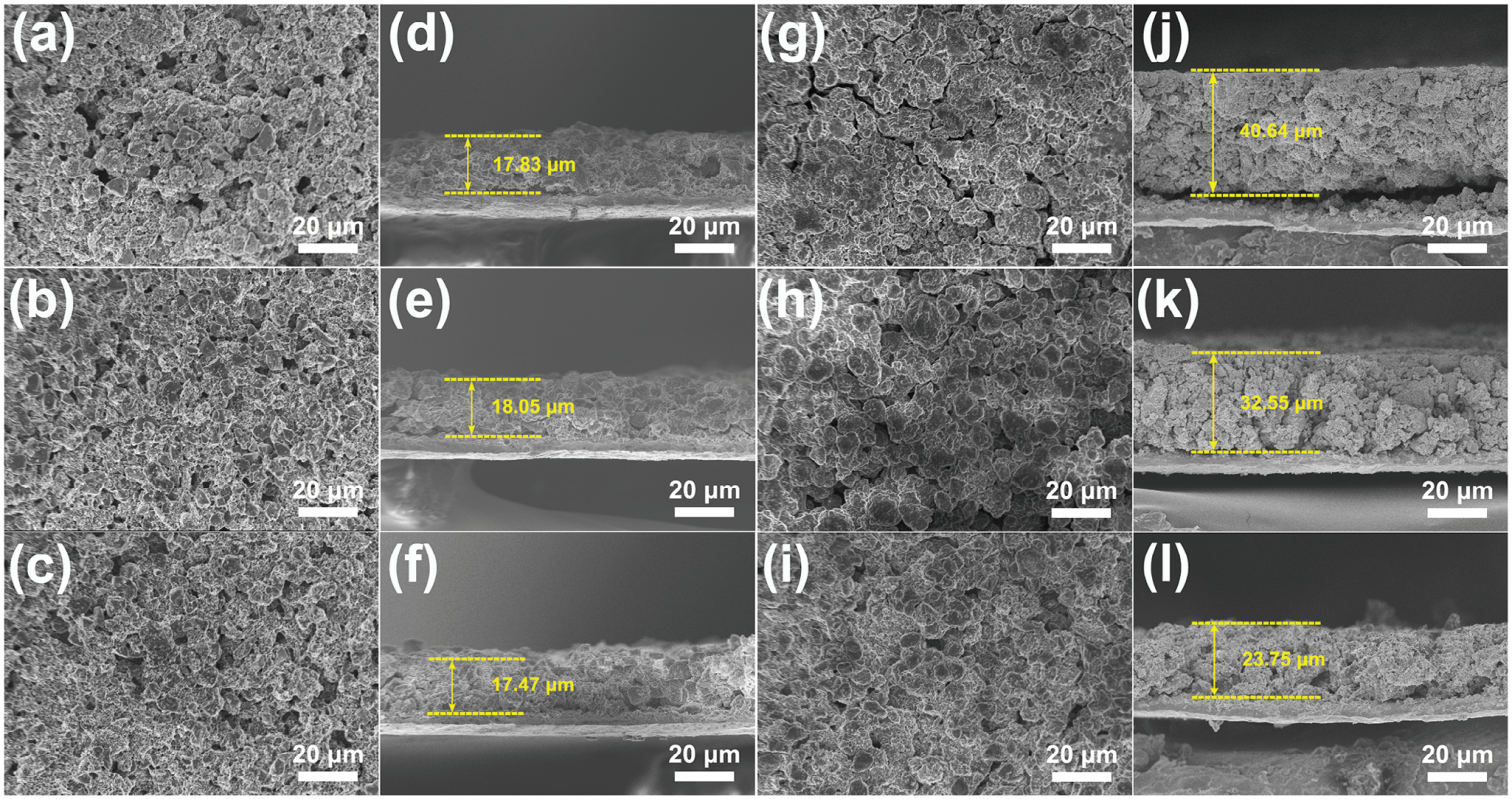
Top‐view SEM images of a) A–SiO*_x_* @C, b) V–SiO*_x_* @C, and c) V–SiO*_x_* @AP@C before cycling. Cross‐sectional SEM images of d) A–SiO*_x_* @C, e) V–SiO*_x_* @C, and f) V–SiO*_x_* @AP@C before cycling. Top‐view SEM images of g) A–SiO*_x_* @C, h) V–SiO*_x_* @C, and i) V–SiO*_x_* @AP@C after 200 cycles. Cross‐sectional SEM images of j) A–SiO*_x_* @C, k) V–SiO*_x_* @C, and l) V–SiO*_x_* @AP@C after 200 cycles.

In order to further certify the stability of V–SiO*_x_* @AP@C electrode with respect to the structure and morphology, the SEM images of A–SiO*_x_* @C, V–SiO*_x_* @C, and V–SiO*_x_* @AP@C electrodes before and after 200 cycles are studied (**Figure** **4**; Figure S18, Supporting Information). As shown in Figure S18a (Supporting Information) and 4a, the surface of the A–SiO*x*@C electrode is inhomogeneous, in which a part has high porosity and a part has relatively dense particle accumulation owing to uneven particle size of the active substance. In contrast, the active substances are evenly distributed on the surface of V–SiO*_x_* @C (Figure 4b; Figure S18b, Supporting Information) and V–SiO*_x_* @AP@C (Figure 4c; Figure S18c, Supporting Information) anodes with uniform pores. After cycling, huge cracks can be observed on the A–SiO*_x_* @C anode, and many particles of A–SiO*_x_* @C stick together to form large divided “island” blocks with no clear boundary between the particles (Figure 4g; Figure S18d, Supporting Information). Cross‐sectional SEM images exhibit that the thickness of the electrode increases from 17.83 to 40.64 µm (Figure 4d–j), accompanied by an increased ratio of up to 128%, and there is a clear separation between the active substances and current collectors. It is precisely because the large silicon grains in A–SiO*_x_* @C cause the aggregation of the Li*_x_* Si domains and release tremendous stress during the charging/discharging process, leading to the serious plastic deformation and shedding of the active substances. As a result, the cycling performance deteriorates seriously.

By comparison, the electrode surfaces of V–SiO*_x_* @C (Figure 4h; Figure S18e, Supporting Information) still maintain flat without cracks after cycling. Although a certain degree of expansion causes V–SiO*_x_* @C to appear as irregular elliptical particles, these particles are not broken and maintain their independent morphologies. The thickness expansion ratio of V–SiO*_x_* @C after cycling greatly decreases compared with that of A–SiO*_x_* @C, which is ≈80% (Figure 4e–k). The fine silicon grains and amorphous SiO*_x_* phases evenly embedding in the V–SiO*_x_* form a novel network of amorphous SiO*_x_* connecting each silicon grain, which effectively homogenizes the Li*_x_* Si domains and alleviates the expansion stress to ensure the electrode integrity. Further, the particles on V–SiO*_x_* @AP@C anode almost maintain the primary morphologies (Figure 4I; Figure S18f, Supporting Information) after cycling, and the corresponding thickness expansion ratio is less than 36% (Figure 4f–l). The results show that the amorphous AlPO_4_ coating plays an important role in inhibiting volume expansion and maintaining electrode integrity. More specifically, at first, the amorphous AlPO_4_ layer provides robust and fast‐ion conducting interphase to facilitate the electron transfer and optimize the storage behavior of lithium ions, ensuring the reversible lithiation/delithiation process. Second, the amorphous AlPO_4_ layer can be used as the protective barrier to reduce direct contact between the electrolyte and the active substance, contributing to the formation of stable SEI and avoiding the plastic deformation of SiO*_x_*. Third, the PO_4_^3−^ in AlPO_4_ has strong covalence with Al^3+^ ion, which can resist chemical attack and enhance the thermal stability of the V–SiO*_x_* @AP@C anode.

The compositions of SEI layers on A–SiO*_x_* @C, V–SiO*_x_* @C, and V–SiO*_x_* @AP@C electrode surface after 100 cycles are investigated by the XPS analysis. The peaks of C, O, F, and Li elements are detected in the XPS survey spectra (Figure S19a, Supporting Information). As shown in Figure S19b (Supporting Information), the peak at 685.0 eV is assigned to the formation of LiF, an important component of SEI, which can build a passivation layer and avoid the further reduction of electrolyte.^[^ ^29^ ^]^ Then, the peak at 687.0 eV corresponds to P─F bonds of the Li*_x_* PO*_y_* F species, which are formed by the electrolyte decomposition. Obviously, the relative intensity of LiF in V–SiO*_x_* @C and V–SiO*_x_* @AP@C is higher than that of A–SiO*_x_* @C, while the relative intensity of the Li*_x_* PO*_y_* F species is opposite, demonstrating less decomposition of electrolyte of V–SiO*_x_* @C and V–SiO*_x_* @AP@C. The large silicon grains in A–SiO*_x_* @C are easy to cause particle breakage during the cycling process of repeated volume expansion, and SEI is constantly broken and regenerated, increasing the consumption of electrolyte. In contrast, the fine grains in V–SiO*_x_* @C and V–SiO*_x_* @AP@C are beneficial to alleviate the volume expansion of the particles, and the particle integrity is more conducive to the formation of stable SEI. The highest relative intensity of LiF in V–SiO*_x_* @AP@C also indicates that the AlPO_4_ protective layer with stable physicochemical and mechanical properties contributes to stabilizing the SEI. In Figure S19c (Supporting Information), the peaks at 55.0 and 55.4 eV belong to Li_2_CO_3_ and LiF.^[^ ^30^ ^]^ Besides, in the C 1s spectra, the peaks at 284.8 and 286.5 are attributed to C─C and C─O bonds and the peaks at 288.3 and 289.8 eV correspond to C═O and the electrolyte decomposition products of ROCO_2_Li/Li_2_CO_3_ (Figure S19d, Supporting Information).^[^ ^29a^ ^]^

**Figure 5:**
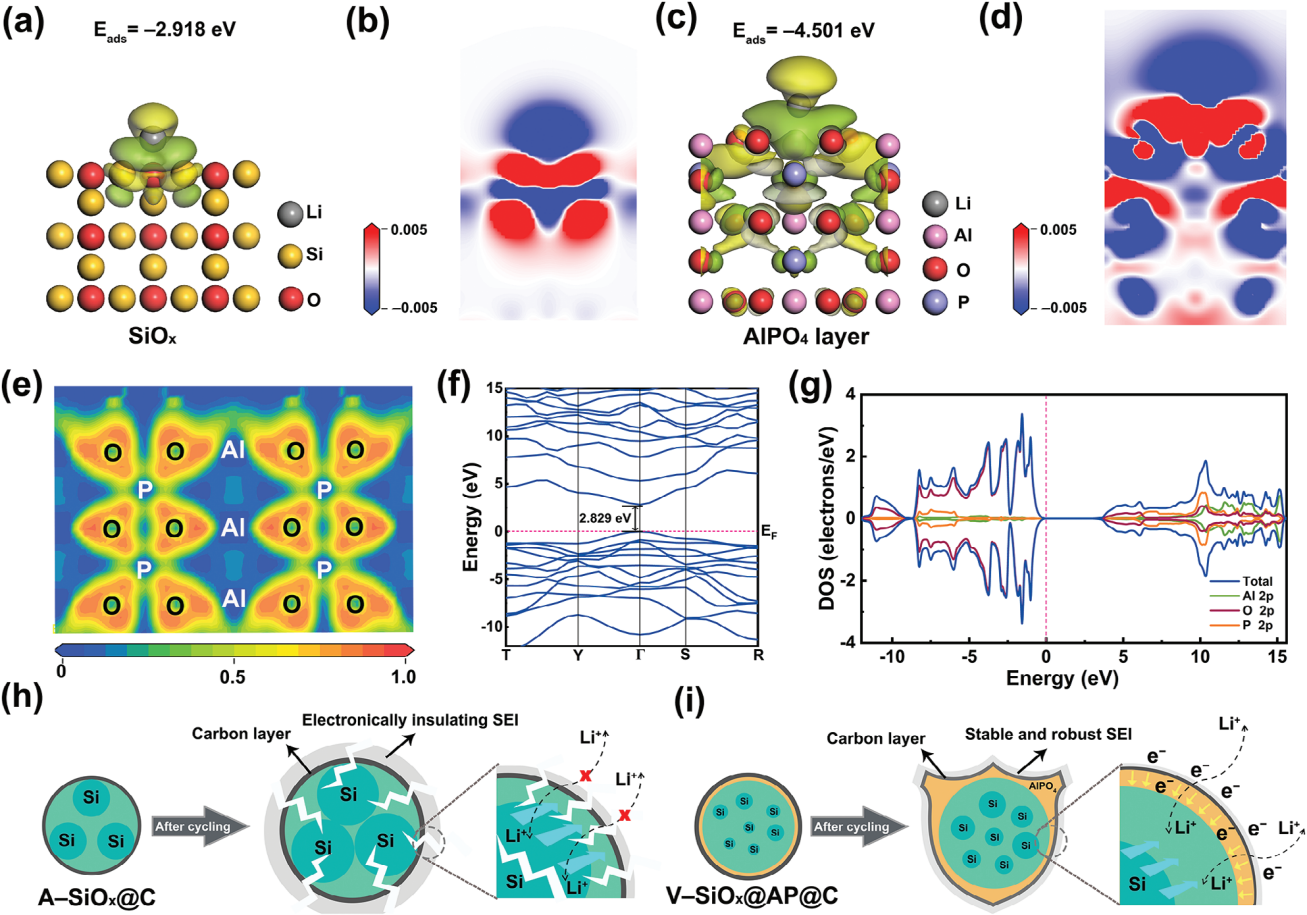
Charge density difference distribution of lithium ions on the a) SiO*_x_* and c) AlPO_4_ layer. Corresponding sliced 2D contour maps of lithium ions on the b) SiO*_x_* and d) AlPO_4_ layer. e) ELF, f) band structure, and g) DOS of AlPO_4_. The schematic diagrams of h) plastic deformation of A–SiO*_x_* @C anode and i) the effect to relieve volume change and improve the ion/electron transport of V–SiO*_x_* @AP@C anode during the cycling process.

The density functional theory (DFT) calculations are performed to further investigate the effect of the AlPO_4_ coating layer by constructing the corresponding molecular models. **Figure** **5**a–d exhibit the differential charge density distributions and correspondingly sliced 2D contour maps of lithium ions on the SiO*_x_* and AlPO_4_ layer. The charge accumulation and depletion can be clearly observed through the molecular models, illustrating the charge transfer between the interfaces of lithium ions and the SiO*_x_* and AlPO_4_ layer. The calculated adsorption energy (*E*_ads_) of lithium ions on the AlPO_4_ layer is −4.501 eV, much higher than that of lithium ions on SiO*_x_* (*E*_ads_ = −2.918 eV), which demonstrates the favored adsorption of lithium ions on the AlPO_4_ layer. Therefore, the AlPO_4_ coating layer can ensure that sufficient lithium ions are adsorbed on the surface of the V–SiO*_x_* @AP@C, thereby promoting the transfer and distribution of lithium ions to optimize the capacity and rate performance of the battery. In addition, there is a strong local positive charge concentration on the AlPO_4_ layer adsorbed with lithium ions, which even affects the charge distribution inside the AlPO_4_ molecular model, further proving the strong adsorption capability of AlPO_4_ to lithium ions. Meanwhile, electron localization function (ELF) can be used as an indicator of bond status between atoms, where the ELF value smaller than 0.5 demonstrates the electron delocalization, corresponding to ionic bonds, while a larger value than 0.5 means stronger electron accumulation ability, indicating the formation of covalent bonds.^[^ ^31^ ^]^ As shown in Figure 5e, the calculated ELF results among Al, O, and P are higher than 0.5, confirming the formation of Al─O─P covalent bonds.^[^ ^32^ ^]^ The covalent bonds not only provide robust structures of the AlPO_4_ layer to maintain the stability of V–SiO*_x_* @AP@C during the repeated cycling process but also offer stable physicochemical properties for the protective layer to avoid the occurrence of side reactions. The Young's modulus distribution of V–SiO*_x_* and V–SiO*_x_* @AP is quantitatively evaluated by atomic force microscope (AFM). As shown in Figure S20 (Supporting Information), with the formation of AlPO_4_ on V–SiO*_x_*, the average Young's modulus is increased from 2.6 to 5.6 GPa, indicating that the introduction of AlPO_4_ coating can effectively control the plastic deformation of SiO*_x_*.

Besides, the band structures and density of states (DOS) of AlPO_4_ are analyzed (Figure 5f,g) to investigate the electronic structure. Valence bands below Fermi energy are mainly formed by O 2p orbitals, while the conduction bands above Fermi energy are mainly formed by Al 2p, O 2p, and P 2p orbitals. Moreover, as the Fermi energy is in the interval where DOS is zero and the valence band maximum and conduction band minimum are located at the same Γ‐point in the Brillouin region, AlPO_4_ is a direct‐gap semiconductor with a small band gap of 2.829 eV. Hence, the valence band electrons of AlPO_4_ can easily jump to the conduction band, forming conductive carriers, which exactly explains the good electrical conductivity of the V–SiO*_x_* @AP@C anode in the above EIS test results.^[^ ^33^ ^]^ The conductive AlPO_4_ coating can better transfer electrons to effectively reduce internal resistance, avoiding capacity loss and improving the cycling performance, which is consistent with the previous electrochemical performance analysis. Based on the above experimental result discussion and theoretical simulation analysis, the evolution of A–SiO*_x_* @C and V–SiO*_x_* @AP@C during the cycling process is illustrated in Figure 5 h,i. As exhibited in Figure 5h, the severe volume change of large silicon grains in A–SiO*_x_* @C will result in material pulverization and thick SEI accumulation, which will deteriorate the ability of A–SiO*_x_* @C to transport electrons and ions. In contrast, the fine silicon grains in V–SiO*_x_* @AP@C can effectively relieve the stress caused by volume expansion (Figure 5i). The robust protective layer of amorphous AlPO_4_ acts as a shield, which not only inhibits the volume expansion of silicon but also avoids the side reaction between electrolyte and active substances to form stable SEI. Combined with the superior adsorption capability of lithium ions and good electrical conductivity of the AlPO_4_ layer, the transport efficiency of electrons and lithium ions of V–SiO*_x_* @AP@C is greatly enhanced, guaranteeing the stability of the battery cycling. In addition, the flocculent carbon layer can also effectively reduce the direct contact between the electrolyte and SiO*_x_*, alleviate the volume expansion of electrode material, and form a continuous conductive network between the particles to improve the electrode reaction kinetics.

**Figure 6:**
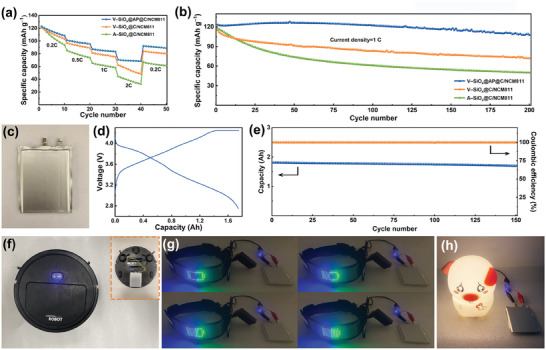
a) Rate performance of A–SiO*_x_* @C/NCM811, V–SiO*_x_* @C/NCM811, and V–SiO*_x_* @AP@C/NCM811 from 0.2 to 2 C (1 C = 200 mA g^−1^). b) Long‐term cycling performance of A–SiO*_x_* @C/NCM811, V–SiO*_x_* @C/NCM811, and V–SiO*_x_* @AP@C/NCM811 at 1 C. c) Photograph and d) typical charge/discharge curves of the V–SiO*_x_* @AP@C–G/NCM811 pouch battery. e) Cycling performance of the V–SiO*_x_* @AP@C–G/NCM811 pouch battery at 0.5 C. Photographs of f) the sweeping robot, g) the light‐emitting diode, and h) the cartoon night light powered by the V–SiO*_x_* @AP@C–G/NCM811 pouch battery.

The full cells are assembled with commercial LiNi_0.8_Co_0.1_Mn_0.1_O_2_ (NCM811) to test the practicability of the prepared materials in LIBs. The rate performance is performed at various current densities from 0.2 to 2C, as shown in **Figure** **6**a (1 C = 200 mA g^−1^). A–SiO*_x_* @C/NCM811 depicts a sharp capacity fading with the increase of current density because the fragmentary particles with large silicon grains are not conducive to relieving the stress and lead to the material pulverization during the cycling process, no longer contributing to the capacity. By contrast, V–SiO*_x_* @C/NCM811 and V–SiO*_x_* @AP@C/NCM811 have higher discharge capacity and more stable capacity retention, benefiting from the electrode integrity and the reinforcement of AlPO_4_ coating. Interestingly, under the high current density of 2C, V–SiO*_x_* @AP@C/NCM811 exhibits significantly improved cycling stability compared with V–SiO*_x_* @C/NCM811, illustrating the positive contribution of the AlPO_4_ coating to good electrical conductivity and robust structure. Meanwhile, V–SiO*_x_* @AP@C/NCM811 also demonstrates excellent long‐term cycling stability, which can deliver a high specific capacity of 107.7 mAh g^−1^ after 200 cycles at 1 C, greatly higher than those of A–SiO*_x_* @C/NCM811 and V–SiO*_x_* @C/NCM811 (Figure 6b). Further, the balance of laboratory research and industrial application is significant to evaluate the practicability of the prepared materials. Therefore, a 1.8 Ah pouch battery is fabricated with V–SiO*_x_* @AP@C–G (V–SiO_x_@AP@C and graphite composite) as the anode and commercial NCM811 as the cathode (Figure 6c). The pouch battery is charged and discharged at 0.5 C between 2.75 and 4.25 V, and the typical charge/discharge curves are exhibited in Figure 6d. In addition, the V–SiO*_x_* @AP@C–G/NCM811 pouch battery delivers an initial capacity of 1.8 Ah and maintains a reversible capacity of 1.7 Ah after 150 cycles (Figure 6e), which demonstrates the extremely great potential in practical application. For example, the V–SiO*_x_* @AP@C–G/NCM811 pouch battery is assembled on the sweeping robot to drive it to sweep the floor (Figure 6f; Videos S1 and S2, Supporting Information). Meanwhile, the V–SiO*_x_* @C–G/NCM811 pouch battery can also be used as a reliable power supply, such as charging the light‐emitting diode (Figure 6g; Video S3, Supporting Information) and powering the cartoon night light (Figure 6h), proving the availability in various application scenarios.

## 3 Conclusion

In this work, the unique V–SiO*_x_* @AP@C anode is successfully prepared and applied in LIBs. The vacuum thermal reduction at low temperature ensures the structural integrity of SiO*_x_* and uniform distribution of fine silicon grains inside, which can effectively avoid material pulverization due to the huge volume expansion. Moreover, the innovative introduction of the multifunctional amorphous AlPO_4_ coating on the V–SiO*_x_* optimizes the reversible kinetics of the anode due to the superior ion/electron conduction properties of AlPO_4_. In addition, the robust AlPO_4_ with strong covalent bonds and stable physicochemical characteristics can act as the protective layer to inhibit the volume change of the anode materials, contributing to prolonging the cycling life of the battery. Consequently, V–SiO*_x_* @AP@C can maintain stable cycling over 200 cycles with an ultrahigh ICE of 87.6% and an initial specific capacity of 1775.8 mAh g^−1^. In addition, the assembled V–SiO*_x_* @AP@C–G/NCM811 pouch battery of 1.8 Ah can also ensure a long‐term cycling life of 150 cycles, demonstrating the potential of the prepared V–SiO*_x_* @AP@C for industrial applications, which is helpful to accelerate the research of silicon‐based anodes in LIBs.

## Conflict of Interest

The authors declare no conflict of interest.

## Supporting information

Supporting Information

Supplemental Video 1

Supplemental Video 2

Supplemental Video 3

## Data Availability

The data that support the findings of this study are available from the corresponding author upon reasonable request.
