# Supplementary material for: Amorphous AlPO4 Layer Coating Vacuum Thermal Reduced SiO x with Fine Silicon Grains to Enhance the Anode Stability
Source: Adv Sci (Weinh). 2024 Jul 30;11(36):2405116. doi: 10.1002/advs.202405116 (PMC11423219; doi:10.1002/advs.202405116)
Supplement: Supplementary file 1 — Supporting Information [file ADVS-11-2405116-s001.docx]

Supporting Information

**Amorphous AlPO_4_ Layer Coating Vacuum Thermal Reduced SiO_x_ with Refined Silicon Grains to Enhance the Anode Stability**

*Jingyi Luan, Hongyan Yuan, Jie Liu*, Naiqin Zhao, Wenbin Hu and Cheng Zhong**

J. Luan, H. Yuan, J. Liu, N. Zhao, W. Hu, C. Zhong

Key Laboratory of Advanced Ceramics and Machining Technology (Ministry of Education), Tianjin Key Laboratory of Composite and Functional Materials, School of Materials Science and Engineering, Tianjin University, Tianjin 300072, China.

E-mail: jieliu0109@tju.edu.cn; cheng.zhong@tju.edu.cn

W. Hu

Joint School of National University of Singapore and Tianjin University,

International Campus of Tianjin University, Binhai New City, Fuzhou 350207, China.

**1. Material preparation**

***1.1 Preparation of thermal reduced SiO_x_ under atmosphere pressure (A–SiO_x_)***

Micrometer-sized silicon monoxide (SiO) and Mg powder were mixed in a molar ratio of 1:1. Then, a certain amount of NaCl was added as a dispersant. The mixture was calcined at 750 °C for 5 h at a rate of 5 °C min^−1^ under an Ar atmosphere and atmospheric pressure. After cooling down to room temperature, the mixture was immersed in 1 M HCl solution to remove MgO and the residual Mg, and then washed with deionized water to completely remove NaCl. After drying, A–SiO_x_ was obtained.

***1.2 Preparation of vacuum thermal reduced SiO_x_ (V–SiO_x_)***

Micrometer-sized SiO and Mg powder with the molar ratio of 1:1 were placed above and under the nickel foam device, respectively. The device was transferred to a tube furnace and then calcined at 750 °C for 5 h at a rate of 5 °C min^−1^ under vacuum. The V–SiO_x_ was obtained after removing MgO and the residual Mg with 1 M HCl solution and deionized water.

***1.3 Preparation of V–SiO_x_ with amorphous AlPO_4_ layer (V–SiO_x_@AP)***

122.8 mg Al(NO)_3_·9H_2_O and 43.2 mg (NH_4_)_2_HPO_4_ were dissolved in deionized water, and then 4 g V–SiO_x_ was dispersed into the above solution, which was stirred at 80 °C until the solvent evaporated completely. Subsequently, the as-prepared samples were annealed at 130 °C for 4h and then 750 °C for 4 h to obtain V–SiO_x_@AP.

***1.4 Preparation of*** ***A–SiO_x_@C, V–SiO_x_@C and V–SiO_x_@AP@C***

A–SiO_x_@C, V–SiO_x_@C and V–SiO_x_@AP@C were obtained by the chemical vapor deposition (CVD) of A–SiO_x_, V–SiO_x_ and V–SiO_x_@AP under a gas mixture of CH_4_ and Ar at 940 °C for 2 h.

**2. Material Characterization**

X-ray diffraction (XRD) patterns were measured using a Bruker D8 X-ray diffractometer with Cu K_α_ radiation. The morphologies and elemental chemical compositions of as-prepared samples were analyzed with a scanning electron microscope (SEM, Hitachi S-4800) and a transmission electron microscope (TEM, JEM-F200) coupled with the Energy Dispersive Spectrometer (EDS). Raman spectra were performed on a Raman microscope (LabRAM HR800). Fourier transform infrared (FTIR) spectroscopy measurements were conducted through a Thermo Scientific Nicolet iS20. X-ray photoelectron spectroscopy (XPS) data were obtained by a Thermo ESCALAB 250XI spectrometer with Al K_α_ radiation. The specific surface areas of the samples were characterized using N_2_ adsorption-desorption isotherms by the Brunauer–Emmett–Teller (BET) method with a volumetric adsorption analyzer (Autosorb-iQ/ASAP 2460). The wettability of electrodes toward electrolyte was tested on the contact angle measuring instrument (DataPhysics DCA 21). The distribution of Young’s modulus on the surface of the samples was detected by an atomic force microscope (AFM, Bruker Dimension ICON).

**3. Electrochemical Measurements**

The electrochemical properties were performed in 2025-type coin cells. All the cells were assembled in an Ar-filled glove box. The anode slurry was composed of 80 wt.% active materials, 10 wt.% Super P and 10 wt.% carboxymethyl cellulose (CMC) + styrene‐butadiene rubber (SBR) binder, which was evenly dispersed in deionized water and then coated on copper foil. The prepared electrode was dried at 80 °C for more than 10 h in a vacuum oven. The cathode slurry was prepared by mixing 80 wt.% LiNi_0.8_Co_0.1_Mn_0.1_O_2_ (NCM811), 10 wt.% Super P and 10 wt.% polyvinylidene fluoride (PVDF) in N-Methyl-2-Pyrrolidone (NMP), which was then painted on aluminum foil. The electrolyte was 1 M LiPF_6_ dissolved in a mixed solvent of ethylene carbonate (EC) and ethyl–methyl carbonate (EMC) (3:7 vol.%) with 10 wt.% fluoroethylene carbonate (FEC).

The half cells were assembled with lithium foil, Celgard-2400 membrane and as-prepared anode as the counter electrode, separator and working electrode, respectively. Galvanostatic charge/discharge cycling performance was tested on a Neware CT-4008Tn-5V10mA-HWX battery testing system between 0.005–1.5 V (vs. Li^+^/Li). Cyclic voltammetry (CV) measurements were performed on a CS310X electrochemical workstation between 0.005 and 1.5 V. Electrochemical impedance spectroscopy (EIS) was performed with the frequency range of 10^5^ to 10^–2^ Hz. For the full cell test, the as-prepared anode and NCM811 cathode were assembled and the N/P ratio (capacity ratio of the negative and positive electrodes) was about 1.1 and the corresponding galvanostatic charge/discharge curves were performed in the voltage range of 2.7 to 4.3 V.

**4. Computational details**

The density functional theory (DFT) calculations were performed by the Materials Studio software using the generalized gradient approximation (GGA) and Perdew–Burke–Ernzerh (PBE) functional for the exchange-correlation energy.^[1]^ The energy cutoff of the plane wave basis was set at 489.8 eV. The convergence tolerance of electronic self-consistent energy and force was 2 × 10^−5^ eV atom^−1^ and 0.05 eV Å^−1^, respectively. The thickness of the vacuum layer was set as 12 Å. 3 × 3 × 3 k-point grid based on Monkhorst-Pack was applied to sample the Brillouin zone and density of states (DOS). In addition, the adsorption energy (*E*_ads_) for the lithium atom is calculated as follows:

*E*_ads_ = *E*_total_ – *E*_ad_– *E*_sub_ (1)

where *E*_total_, *E*_ad_ and *E*_sub_ refer to the total energy of the lithium atom combined with the substrate, the energy of the adsorbate and the energy of the substrate, respectively.

**Supporting Figures**

**
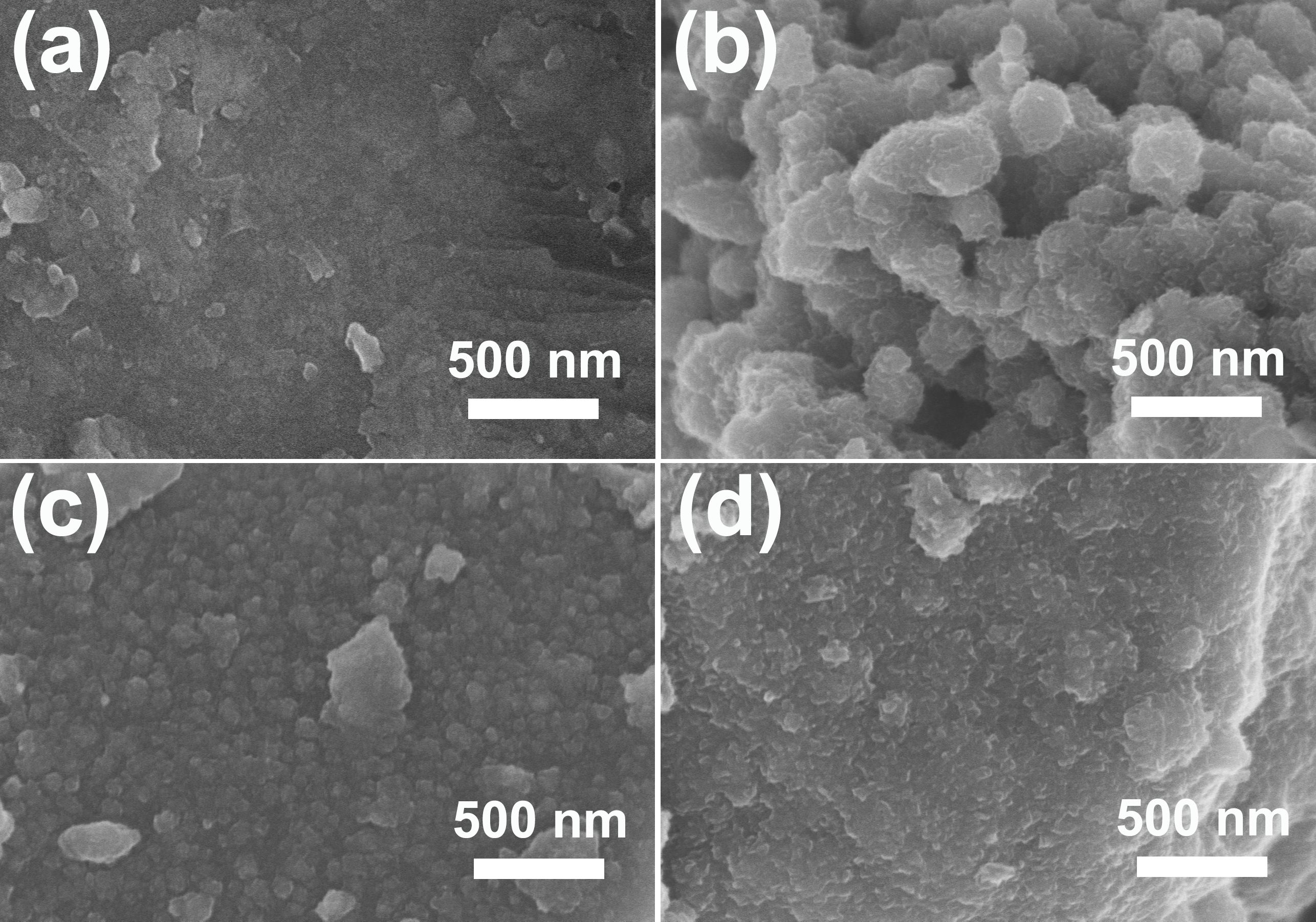
**

**Figure S1** Magnified SEM images of a) SiO, b) A–SiO_x_@C, c) V–SiO_x_@C and d) V–SiO_x_@AP@C.

**
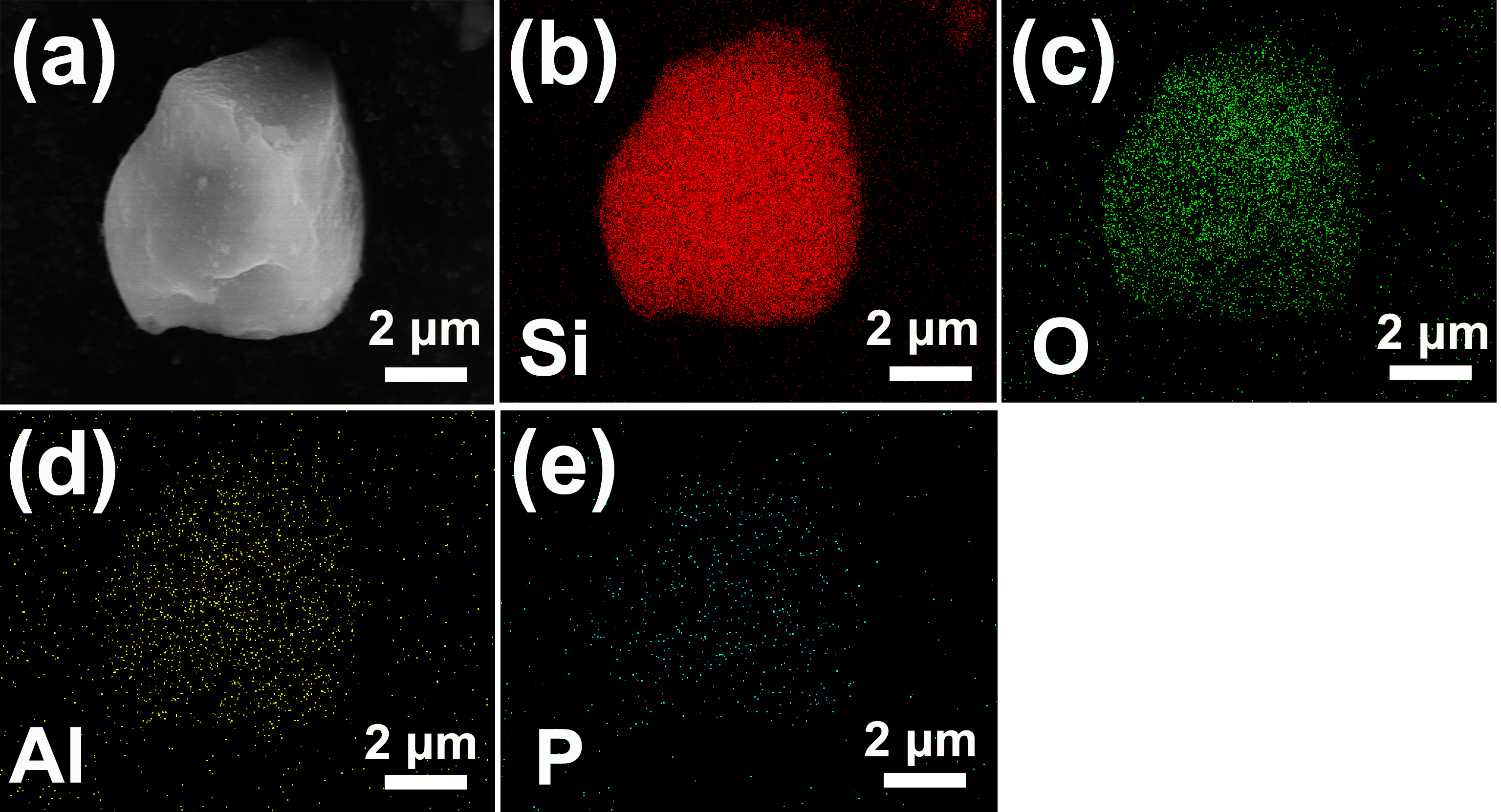
**

**Figure S2** a) SEM image of a single particle of V–SiO_x_@AP and b–e) the corresponding elemental mapping images of Si, O, Al and P.

**
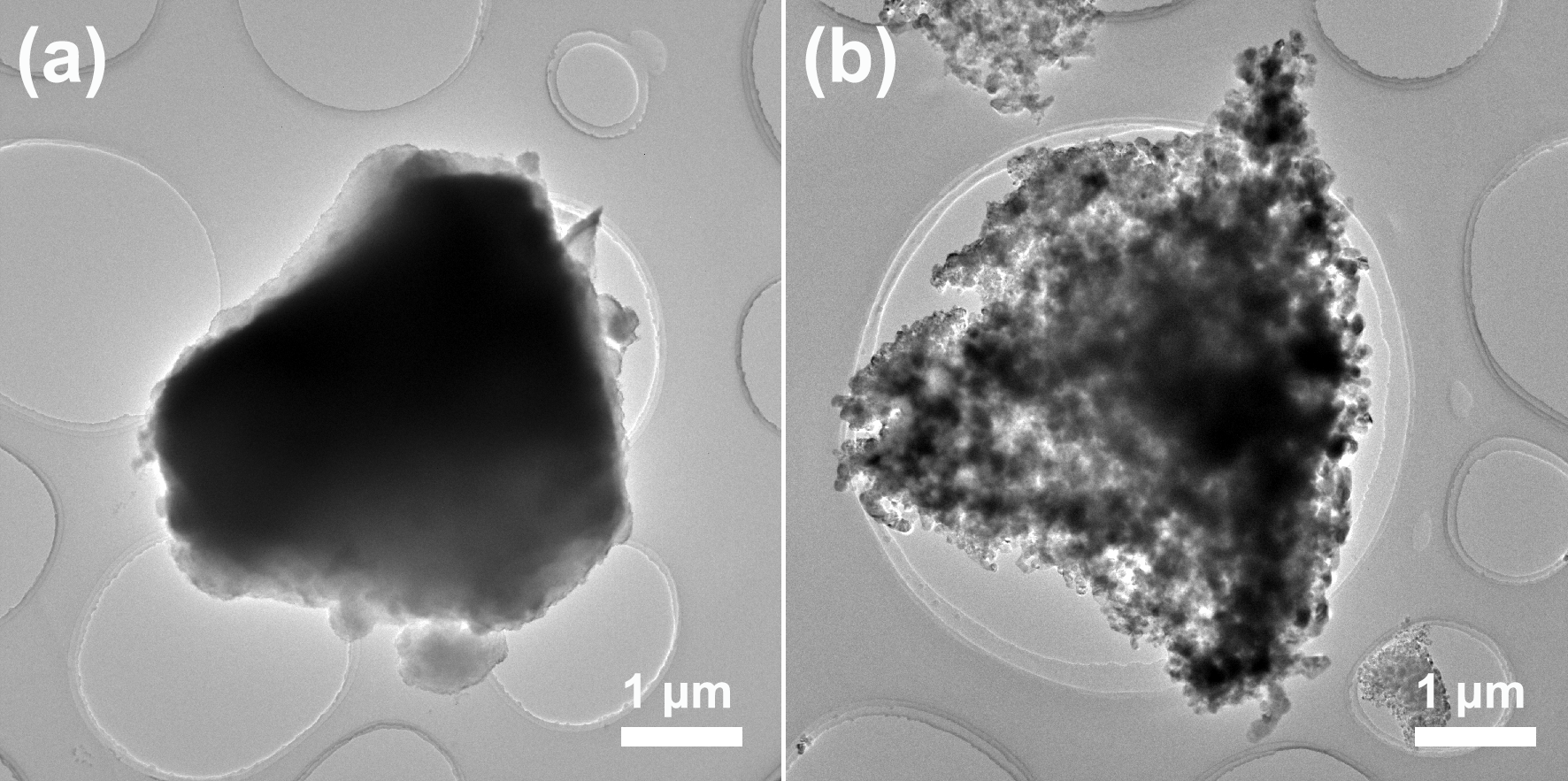
**

**Figure S3** TEM images of a) SiO and b) A–SiO_x_ at low magnification.

**
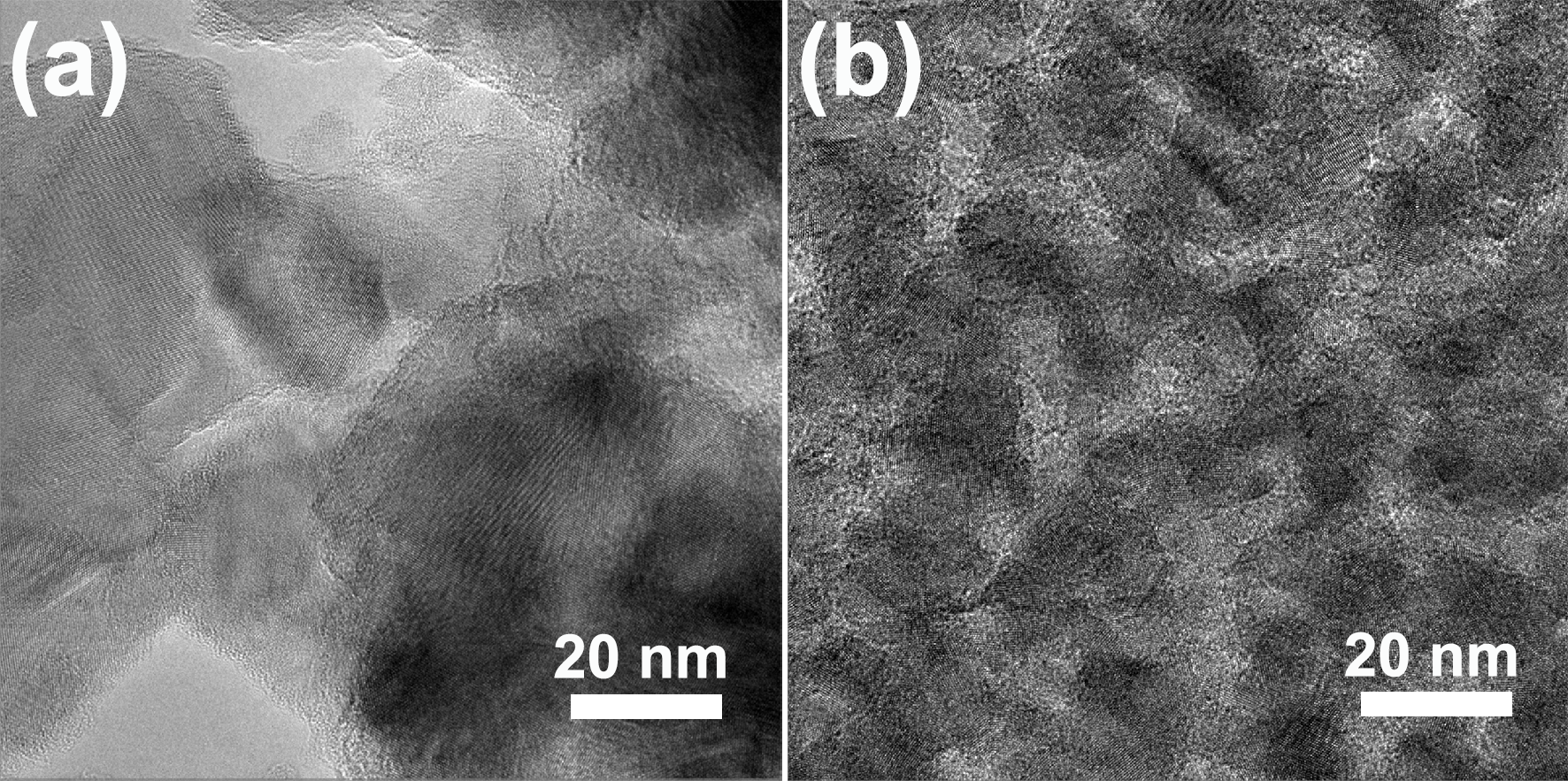
**

**Figure S4** HRTEM images of a) A–SiO_x_ and b) V–SiO_x_.


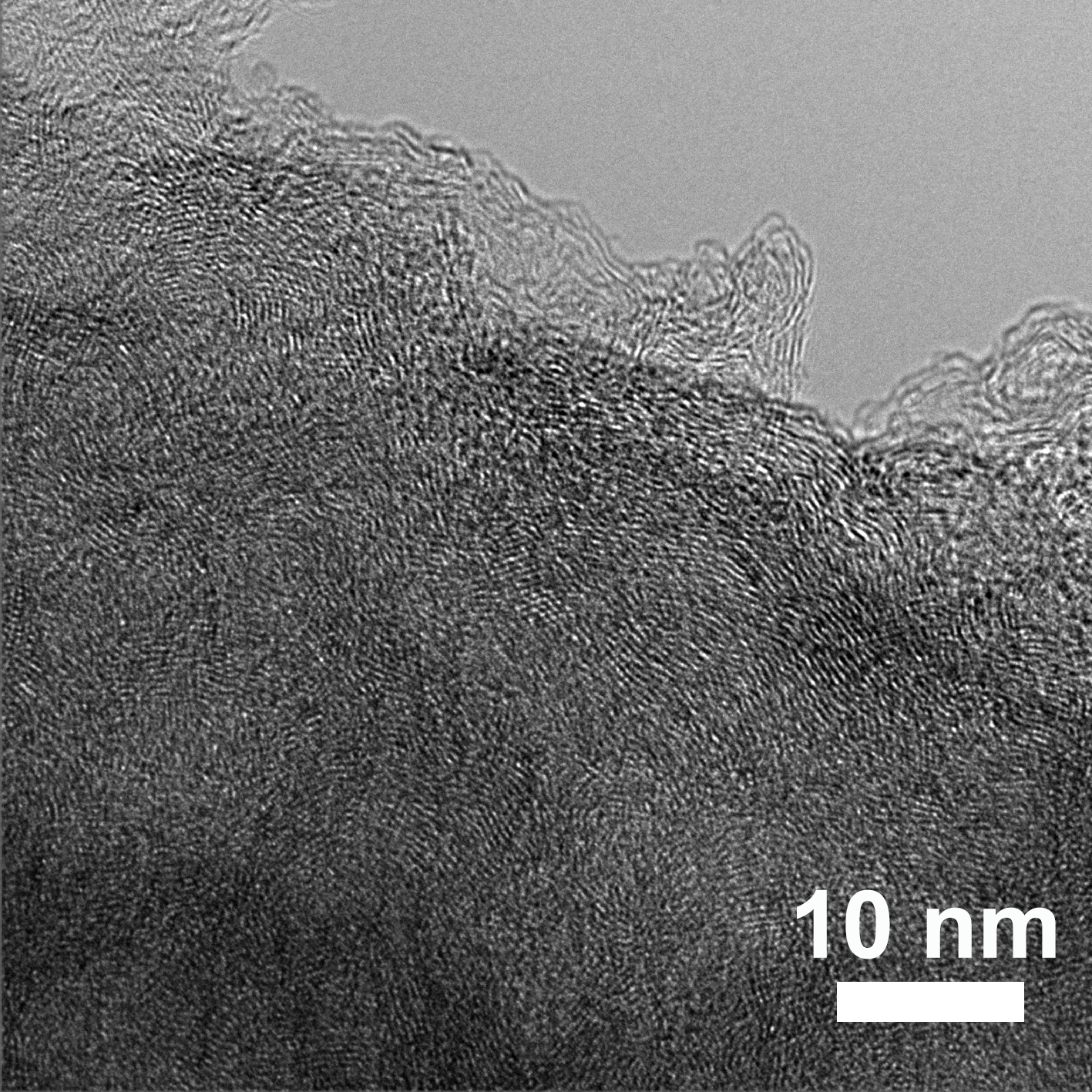


**Figure S5** HRTEM image of the carbon layer of V–SiO_x_@AP@C.





**Figure S6** XRD pattern of pristine micrometer-sized SiO.


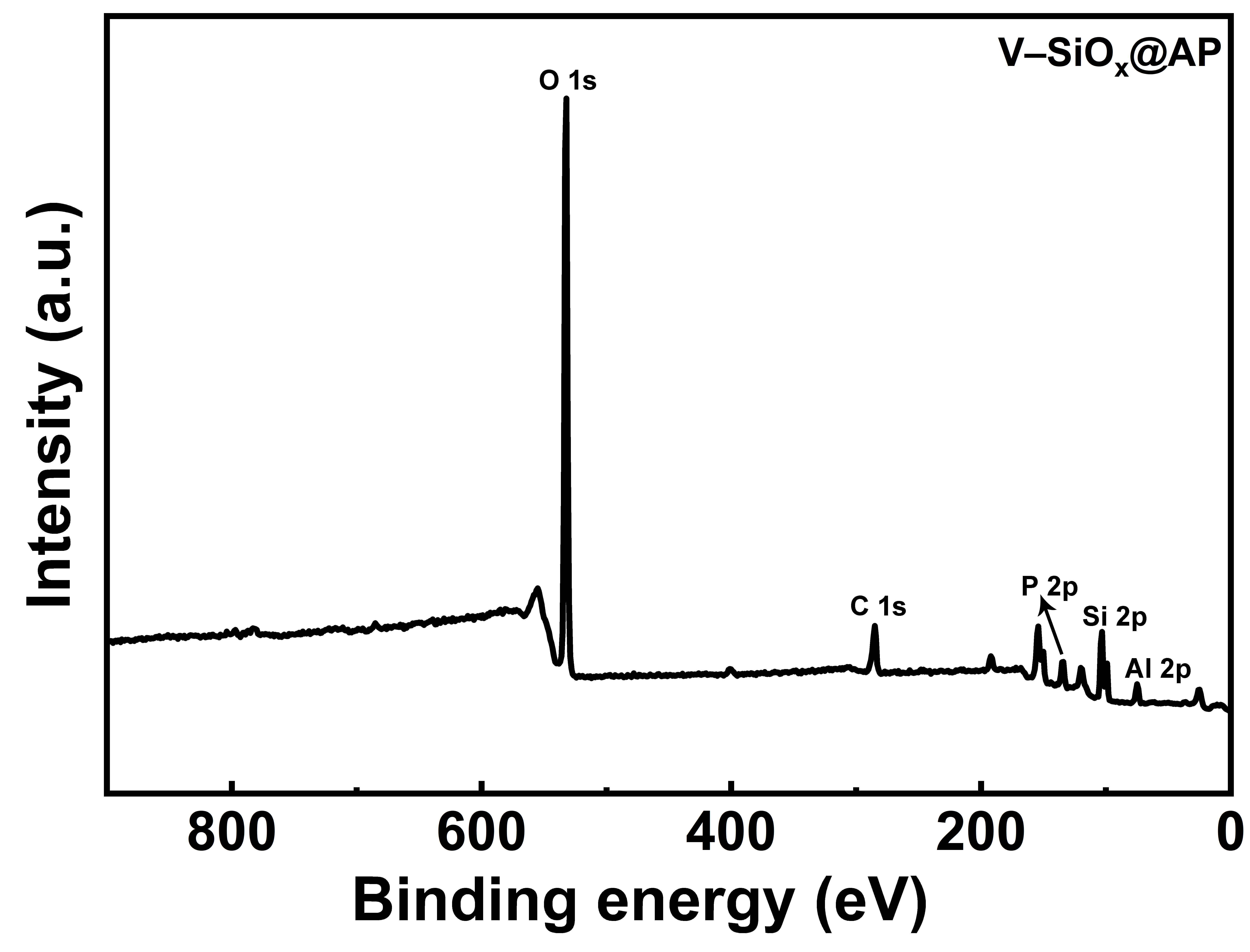


**Figure S7** XPS survey spectrum of V–SiO_x_@AP.





**Figure S8** N_2_ adsorption–desorption isotherms and the corresponding pore size distribution (inset) of a) SiO and b) V–SiO_x_@C.

**
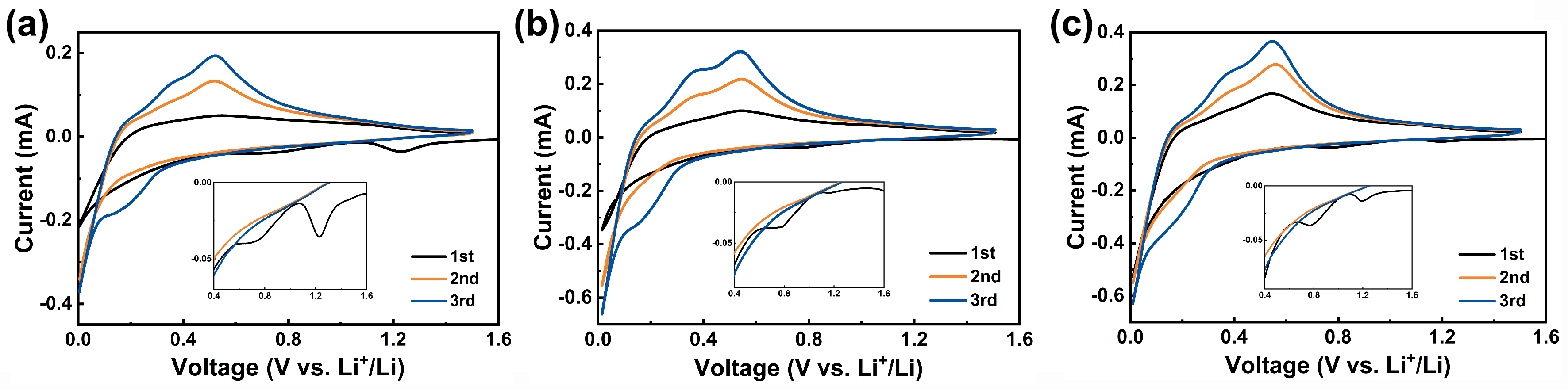
**

**Figure S9** CV curves of a) A–SiO_x_@C, b) V–SiO_x_@C and c) V–SiO_x_@AP@C (the inset is the partially enlarged reduction peaks).

**
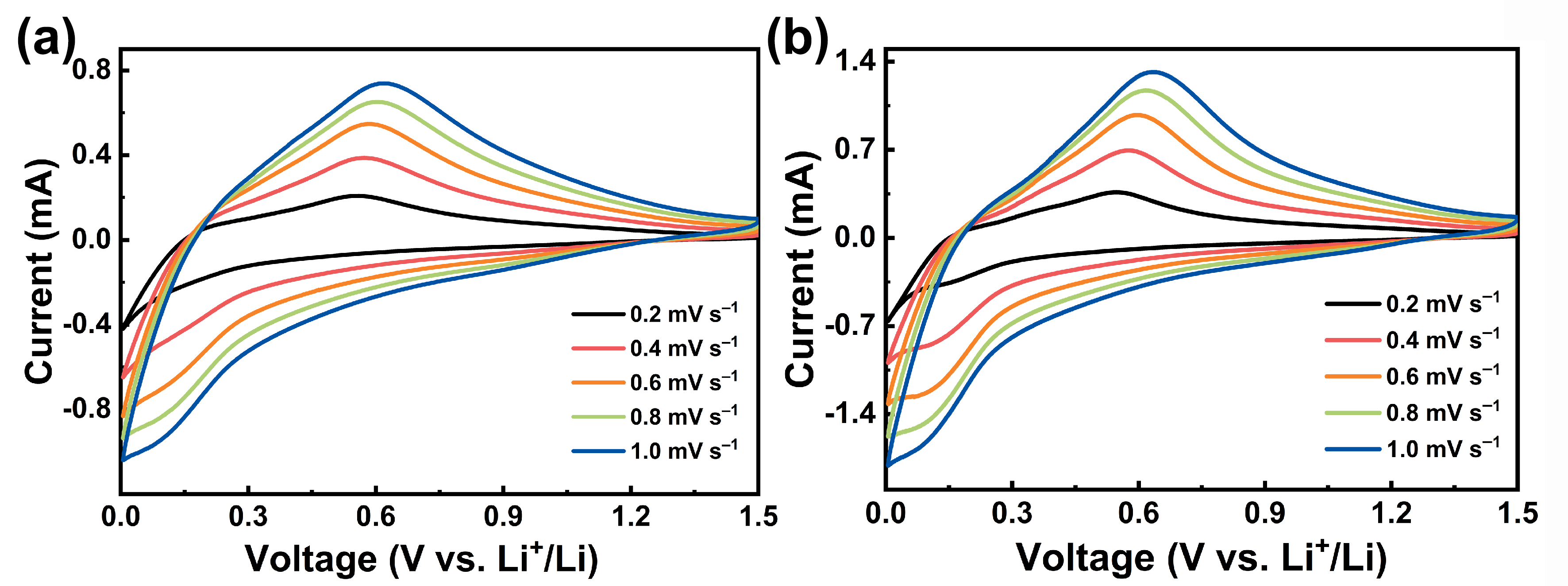
**

**Figure S10** CV curves of a) A–SiO_x_@C and b) V–SiO_x_@C at different scanning rates.


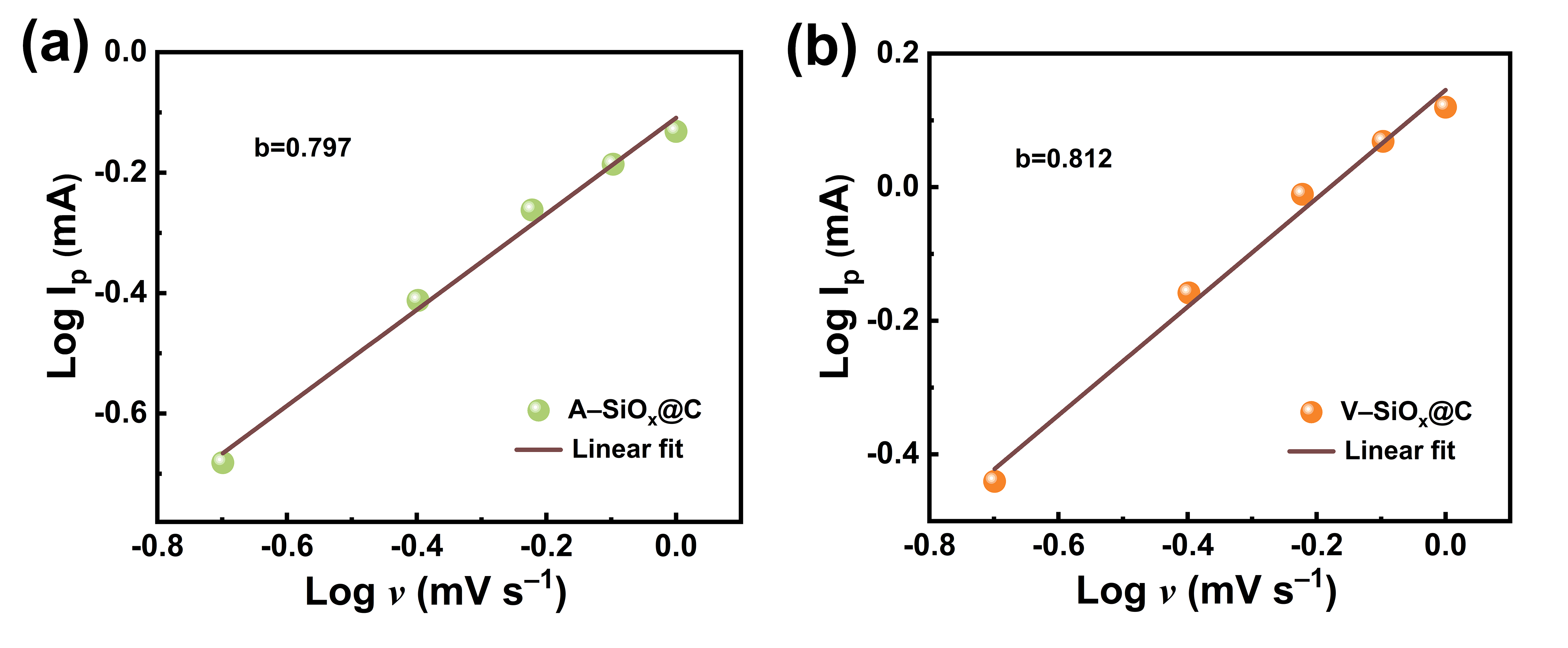


**Figure S11** Logarithm plots of anodic peak currents of a) A–SiO_x_@C and b) V–SiO_x_@C versus scan rates.


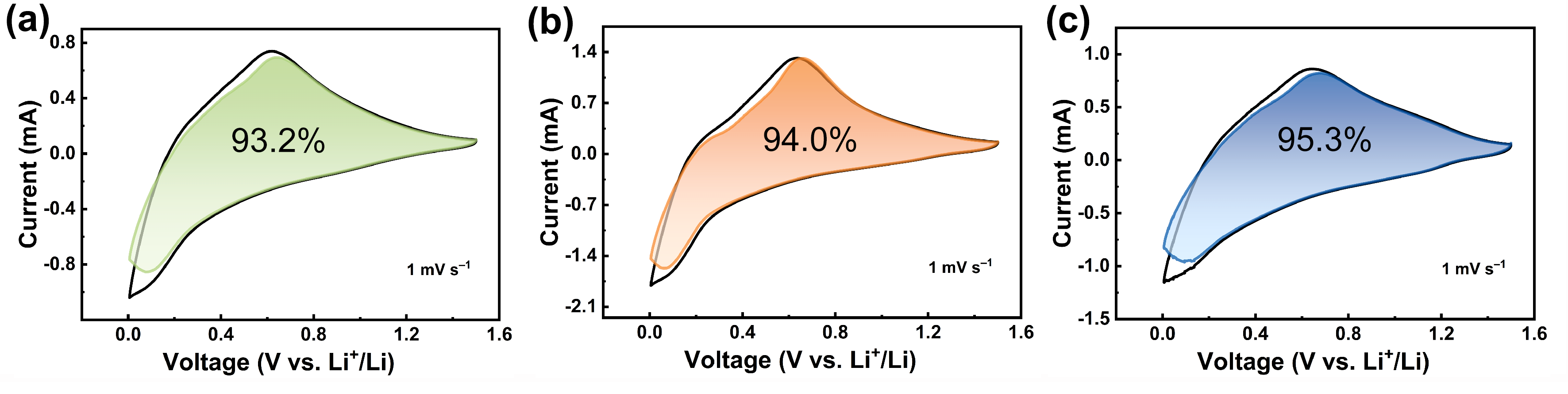


**Figure S12** Percentage capacitive contributions of a) A–SiO_x_@C, b) V–SiO_x_@C c) and V–SiO_x_@AP@C at 1 mV s^−1^.

**
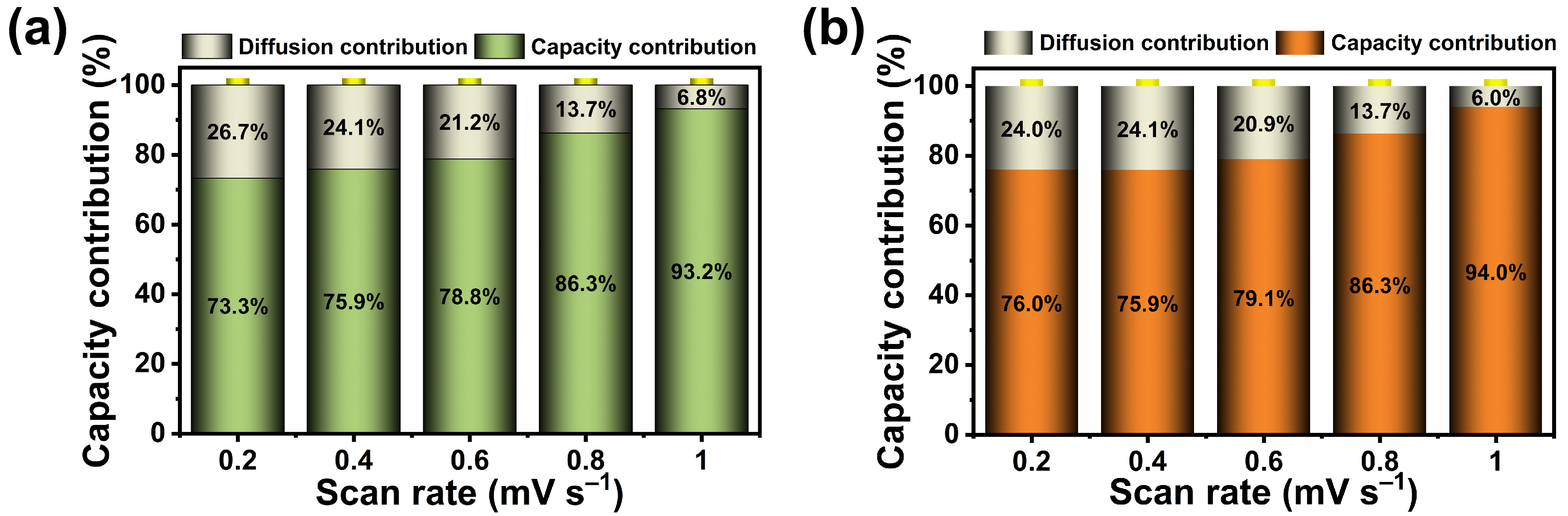
**

**Figure S13** Percentage capacitive contributions of a) A–SiO_x_@C and b) V–SiO_x_@C at different scan rates.

**
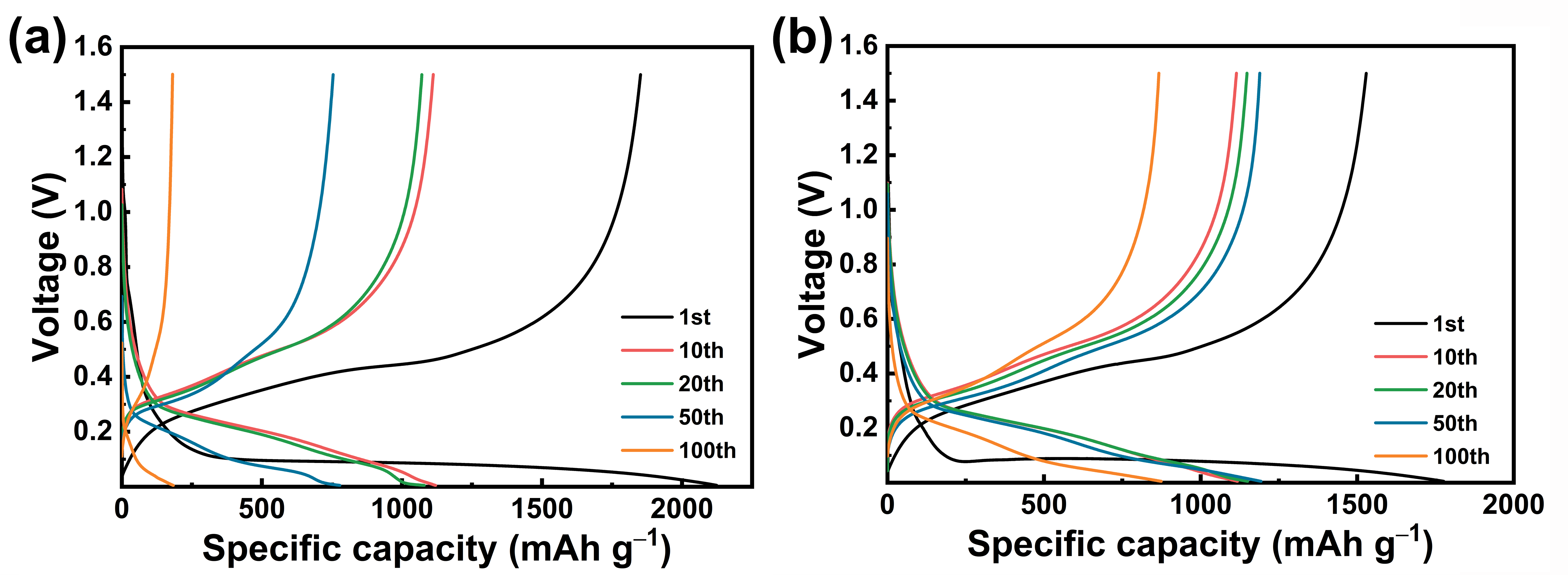
**

**Figure S14** Charge/discharge profiles of (a) A–SiO_x_@C and (b) V–SiO_x_@C with different cycles (the current density of the first two activate cycles is 137.5 mA g^−1^ and the current density of the subsequent cycles is 1 A g^−1^).


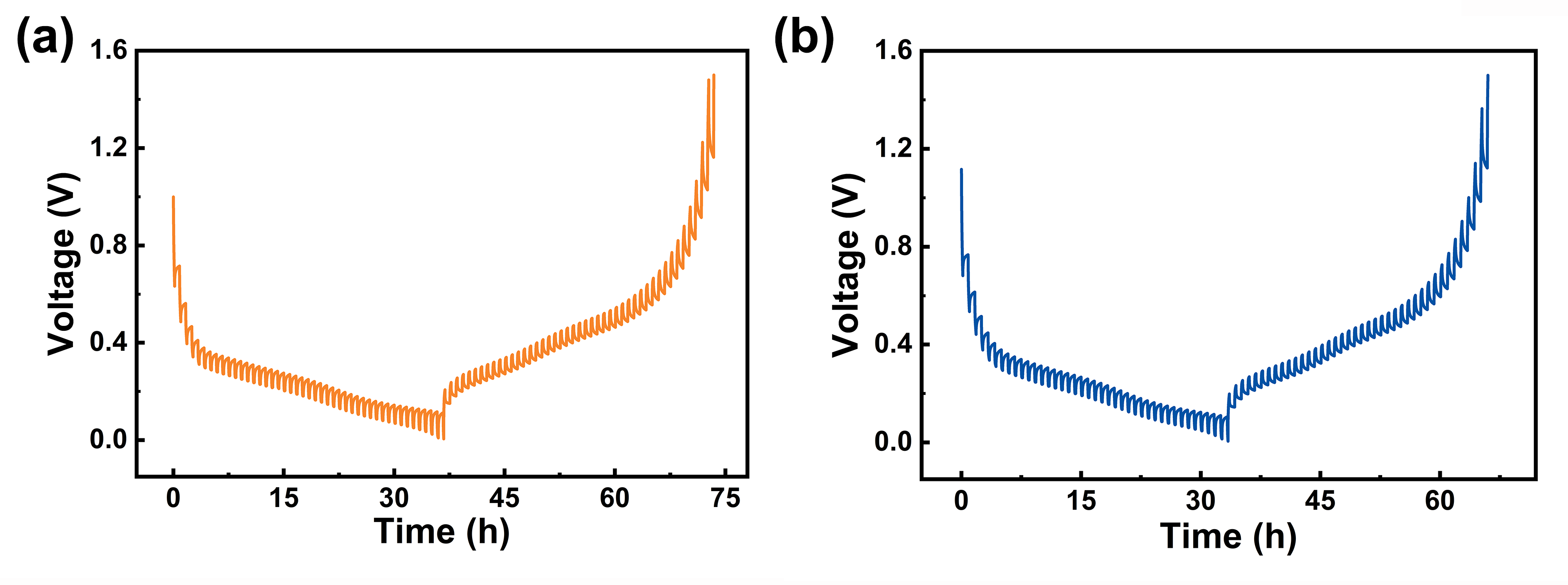


**Figure S15** Galvanostatic intermittent titration technique (GITT) curves (voltage vs time) of a) V–SiO_x_@C and b) V–SiO_x_@AP@C.





**Figure S16** $D_{\mathrm{Li}^{+}}$ against voltage for V–SiO_x_@C and V–SiO_x_@AP@C lithiation process via GITT test.

**
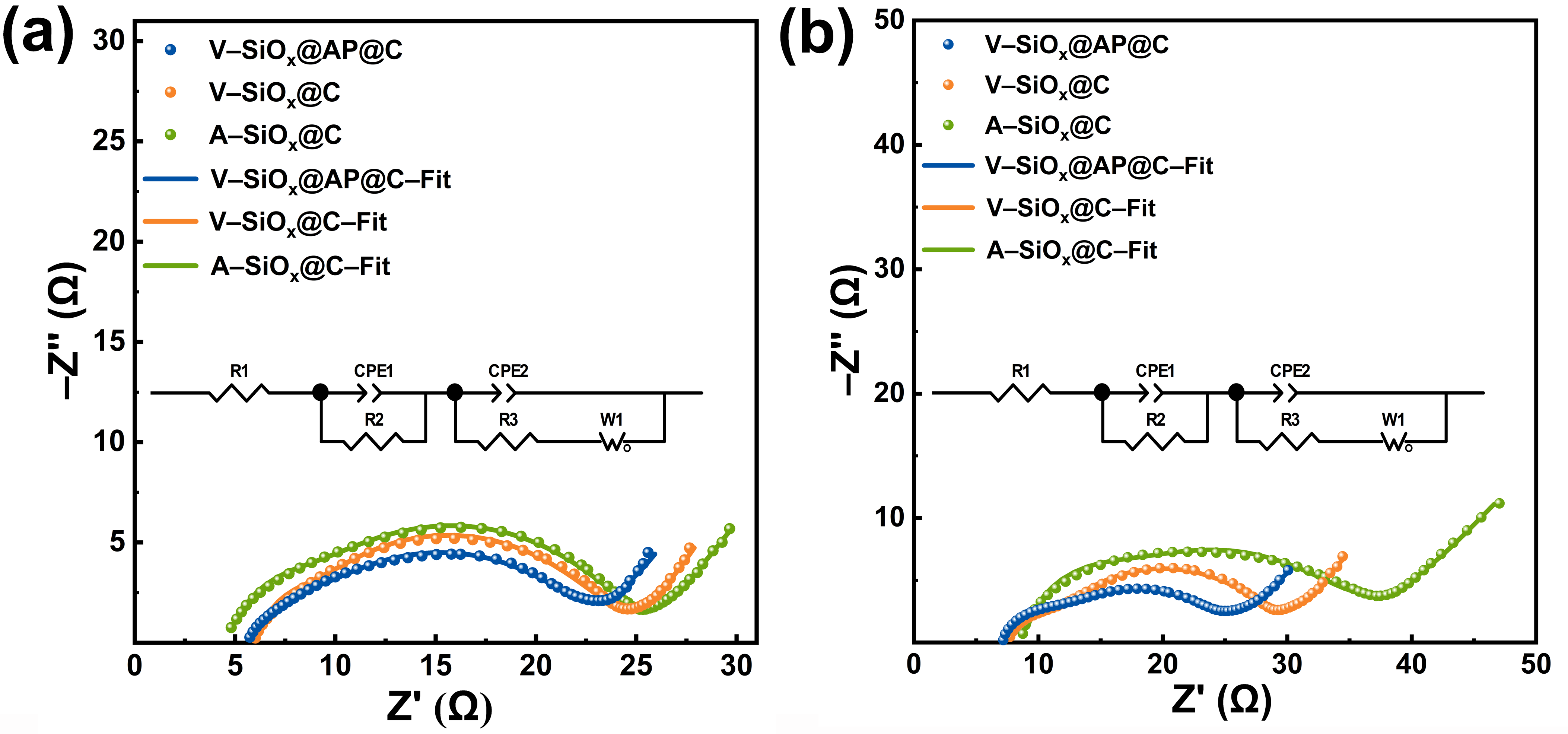
**

**Figure S17** EIS curves of A–SiO_x_@C, V–SiO_x_@C and V–SiO_x_@AP@C a) after 10 and b) 200 cycles.


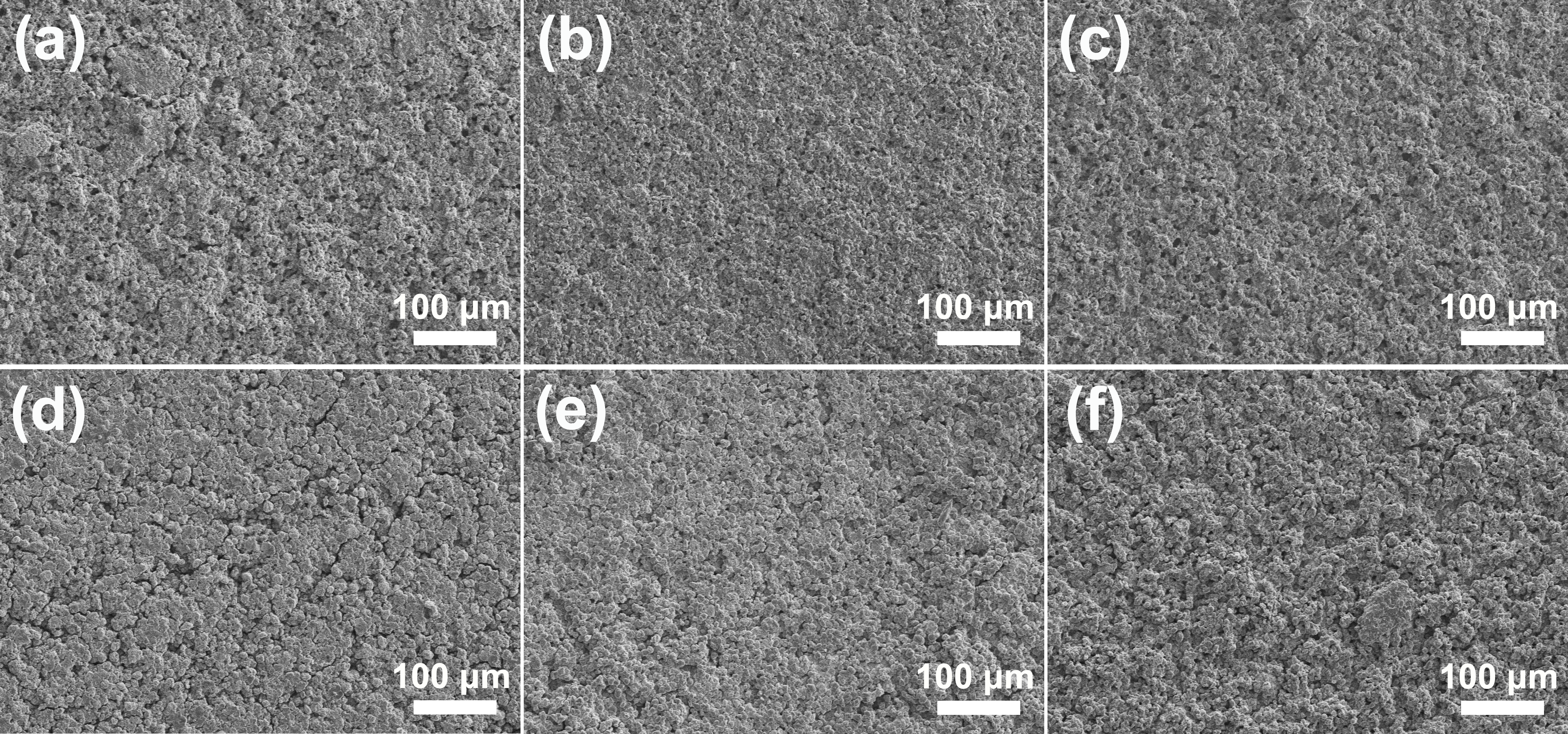


**Figure S18** Top-view SEM images of a) A–SiO_x_@C, b) V–SiO_x_@C, c) V–SiO_x_@AP@C before cycling at low magnification. Top-view SEM images of d) A–SiO_x_@C, e) V–SiO_x_@C, f) V–SiO_x_@AP@C after 200 cycles at low magnification.


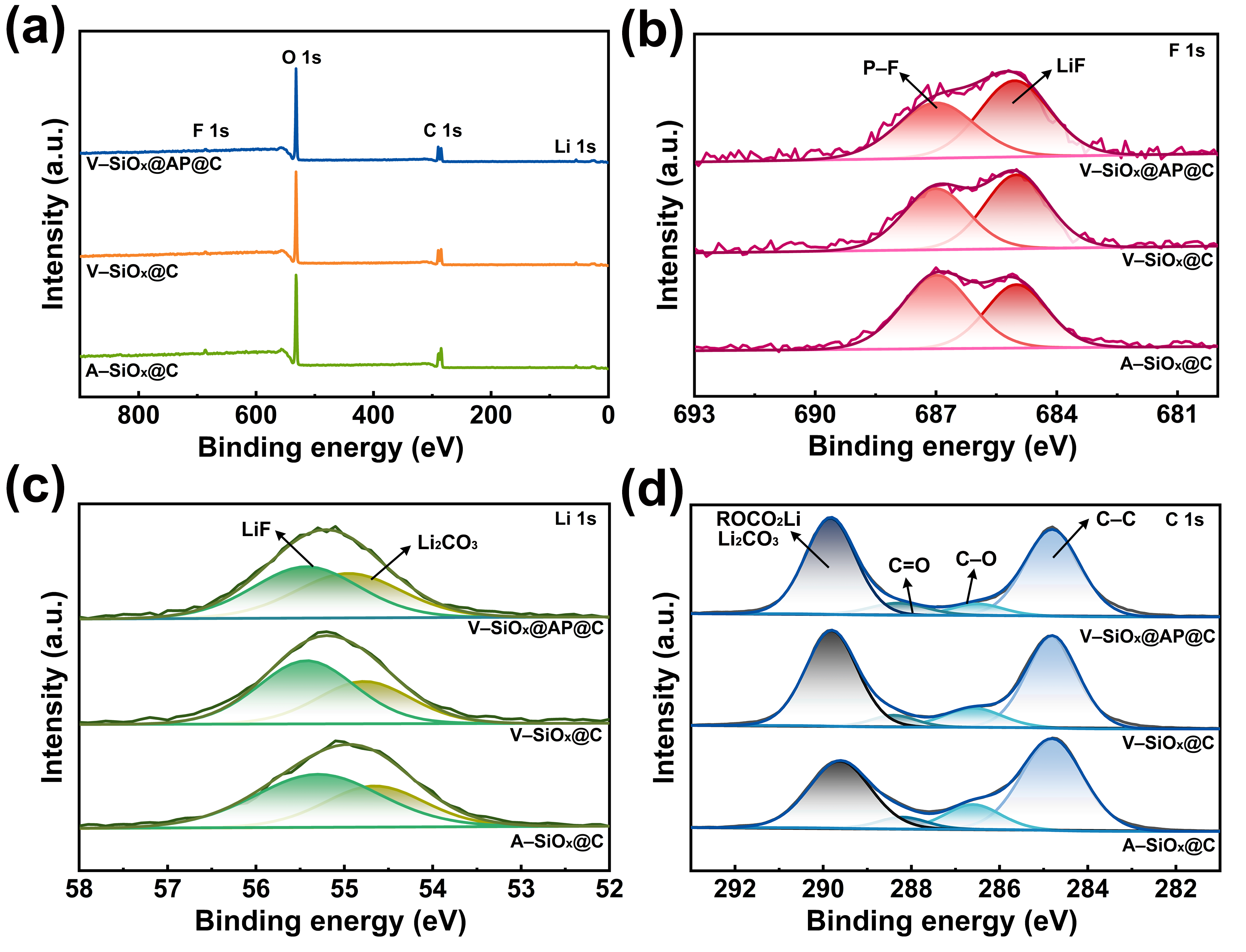


**Figure S19** XPS spectra of (a) survey spectra, (b) F 1 s, (c) Li 1 s and (d) C 1 s of A–SiO_x_@C, V–SiO_x_@C and V–SiO_x_@AP@C electrodes after 100 cycles.


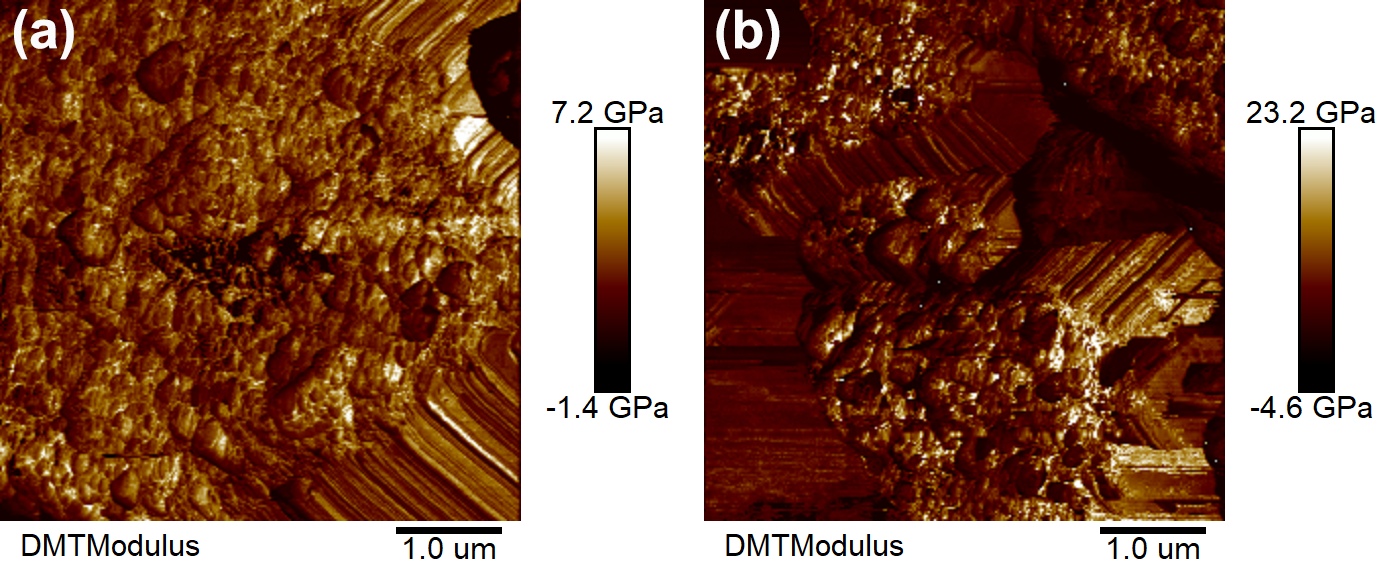


**Figure S20** The distribution of Young’s modulus on the surface of a) V–SiO_x_ and b) V–SiO_x_@AP.

**Table S1** Comparison of the performance of V–SiO_x_@AP@C prepared in this work with previously reported SiO_x_-based anode materials.

| Electrode | Initial Coulombic efficiency (%) | Initial discharge capacity (mAh g^−1^) | Current density (mA g^−1^) | Capacity  retention (100th cycle) | Ref. |
| --- | --- | --- | --- | --- | --- |
| nano-Si/SiO_x_/graphite | 66.0 | 1516 | 100 | 46.8% | [2] |
| SiO_x_–C | ~67.0 | ~1200 | 100 | 56.2% | [3] |
| SiO–SiO_2_@C | ~75.7 | 1566.6 | 200 | 67.7% | [4] |
| MS-800 | 77.2 | 938.6 | 300 | 77.9% | [5] |
| C–SiO–MgSiO_3_–Si-1100 | 78.3 | 1608 | 150 | 60.0% | [6] |
| MSO@C | 81.7 | 1306.1 | 240 | ~62.0% | [7] |
| PBC-0.6 | 82.6 | 994.6 | 400 | 67.2% | [8] |
| Mg13-SiO_x_/C | 87.4 | 1089.7 | 109 | ~36.7% | [9] |
| V–SiO_x_@AP@C | 87.6 | 1775.8 | 1000 | 62.1% | This work |

**References**

[1] W. Zhang, J. Yin, P. Zhang, X. Tang, Y. Ding, *J. Mater. Chem. A* **2018**, *6*, 12029-12037.

[2] C. Park, W. Choi, Y. Hwa, J. Kim, G. Jeong, H. Sohn, *J. Mater. Chem.* **2010**, *20*, 4854-4860.

[3] W. Wu, J. Shi, Y. Liang, F. Liu, Y. Peng, H. Yang, *Phys. Chem. Chem. Phys.* **2015**, *17*, 13451-13456.

[4] X. Hu, P. Xu, M. Liao, X. Lu, G. Shen, C. Zhong, M. Zhang, Q. Huang, Z. Su, *ACS Appl. Energy Mater.* **2024**, *7*, 774-784.

[5] T. Wang, Z. Chen, D. Chen, R. Zhao, *J. Alloys Compd.* **2022**, *899*, 163251.

[6] Y. Zhang, G. Guo, C. Chen, Y. Jiao, T. Li, X. Chen, Y. Yang, D. Yang, A. Dong, *J. Power Sources* **2019**, *426*, 116-123.

[7] C. Bian, R. Fu, Z. Shi, J. Ji, J. Zhang, W. Chen, X. Zhou, S. Shi, Z. Liu, *ACS Appl. Mater. Interfaces* **2022**, *14*, 15337-15345.

[8] X. Li, Z. Yan, S. Yi, J. Jiang, D. Yang, N. Du, *J. Power Sources* **2023**, *570*, 233021.

[9] J. Han, S. Jo, I. Na, S. Oh, Y. Jeon, J. Park, B. Koo, H. Hyun, S. Seo, D. Lee, H. Kim, J. Kim, J. Lim, J. Lim, *ACS Appl. Mater. Interfaces* **2021**, *13*, 52202-52214.
